# Soil microbiota influences clubroot disease by modulating *Plasmodiophora brassicae* and *Brassica napus* transcriptomes

**DOI:** 10.1111/1751-7915.13634

**Published:** 2020-07-19

**Authors:** Stéphanie Daval, Kévin Gazengel, Arnaud Belcour, Juliette Linglin, Anne‐Yvonne Guillerm‐Erckelboudt, Alain Sarniguet, Maria J. Manzanares‐Dauleux, Lionel Lebreton, Christophe Mougel

**Affiliations:** ^1^ INRAE Agrocampus Ouest Université de Rennes IGEPP Le Rheu F‐35650 France; ^2^ INRIA Université Rennes CNRS IRISA Rennes F‐35000 France; ^3^ INRAE Agrocampus Ouest Université de Rennes IGEPP Ploudaniel F‐29260 France; ^4^ INRAE Agrocampus Ouest Université d'Angers IRHS Beaucouzé F‐49071 France

## Abstract

The contribution of surrounding plant microbiota to disease development has led to the ‘pathobiome’ concept, which represents the interaction between the pathogen, the host plant and the associated biotic microbial community, resulting or not in plant disease. The aim herein is to understand how the soil microbial environment may influence the functions of a pathogen and its pathogenesis, and the molecular response of the plant to the infection, with a dual‐RNAseq transcriptomics approach. We address this question using *Brassica napus* and *Plasmodiophora brassicae*, the pathogen responsible for clubroot. A time‐course experiment was conducted to study interactions between *P. brassicae*, two *B. napus* genotypes and three soils harbouring high, medium or low microbiota diversities and levels of richness. The soil microbial diversity levels had an impact on disease development (symptom levels and pathogen quantity). The *P. brassicae* and *B. napus* transcriptional patterns were modulated by these microbial diversities, these modulations being dependent on the host genotype plant and the kinetic time. The functional analysis of gene expressions allowed the identification of pathogen and plant host functions potentially involved in the change of plant disease level, such as pathogenicity‐related genes (NUDIX effector) in *P. brassicae* and plant defence‐related genes (glucosinolate metabolism) in *B. napus*.

## Graphical abstract

The objective was to further the understanding of the complex interaction between the soil microbiota, a plant pathogen and the host plant. The effect of different soil microbial diversities on the level of disease and on the transcriptomes of the interacting pathogen and host plant was studied.

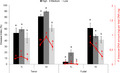

## Introduction

Plants are constantly interacting with a wide variety of potential pathogens within their environment that can cause serious diseases affecting agriculture. The development of biotic plant diseases depends also on the interaction of both plant and pathogen with the environment. All plant tissues, including leaves (Ploch *et al*., 2016; Vacher *et al*., 2016), seeds (Barret *et al*., 2016) and roots (Lundberg *et al*., 2012) are indeed associated with a multitude of microorganisms assembled in microbial communities or microbiota. The complex plant‐associated microbial community structure and composition, as well as the complex network of interactions between microbial species, are crucial in stress tolerance (Rolli *et al*., 2015), plant development dynamics (Chaparro *et al*., 2014), yield, nutrition and health (Mendes *et al*., 2011; Berendsen *et al*., 2012; Badri *et al*., 2013; van der Heijden and Hartmann, 2016). This recognition that the plant microbiota may modulate substantially the disease severity and development led to the ‘pathobiome’ postulation, which refers to the pathogenic agent, its surrounding biotic microbial community and their interactions leading to plant disease (Vayssier‐Taussat *et al*., 2014; Brader *et al*., 2017).

In plants, three root‐associated microbiota compartments can have a role in the modulation of disease development: the soil microbiota, which represents a great reservoir of biological diversity (Raaijmakers *et al*., 2008), the rhizosphere corresponding to the narrow zone surrounding and influenced by plant roots (Mendes *et al*., 2013; Muller *et al*., 2016) and the endosphere (root interior) in which the microbiota diversity is lower than that estimated outside the root (Bulgarelli *et al*., 2012; Turner *et al*., 2013; Vandenkoornhuyse *et al*., 2015; Hassani *et al*., 2018). Several studies have established close relationships between the rhizosphere microbiome composition and the plant immune system (Lebeis *et al*., 2015; Hacquard *et al*., 2017; Bakker *et al*., 2018; Vannier *et al*., 2019), the host genotype resistant or susceptible to a pathogen (Yao and Wu, 2010), and the life‐history traits of bioagressors (Lachaise *et al*., 2017), but the mechanisms underlying these relationships have still to be deciphered. It is also known that plants select microbial communities around their roots by specific root exudates (Yuan *et al*., 2018), that can also function as an additional layer of defence (Berendsen *et al*., 2012). The defence barrier constituted by recruited microorganisms can be of different types: stimulation of defence‐related compounds’ production by the plant, direct antagonism against pathogen (production of antibiotics or antifungal compounds), competition with pathogen for resources (Raaijmakers *et al*., 2008). The invasion by a soil‐borne pathogen led to changes in indigenous plant‐associated microbial communities (Erlacher *et al*., 2014; Lebreton *et al*., 2019) and then in the defence barrier.

Among biotic stress factors, the soil‐borne plant pathogens cause major yield or quality loss in agricultural crops. This is the case of the protist *Plasmodiophora brassicae,* an obligate biotroph responsible for clubroot, one of the economically most important diseases of Brassica crops in the world (Dixon, 2009). The life cycle of this soil‐borne pathogen can be divided into several phases: survival in soil as spores, root hair infection and cortical infection (Kageyama and Asano, 2009). Briefly, during the primary phase of infection, the resting spores germinate in the soil leading to biflagellate primary zoospores that infect the root hairs. In these cells, zoospores multiply to form the primary plasmodia. Secondary zoospores are then released and produce the secondary phase of infection that occurs in the cortex of the roots of the infected plants. During the second phase, multinucleate plasmodia cause the hypertrophy (abnormal cell enlargement) and hyperplasia (uncontrolled cell division) of infected roots into characteristic clubs (Tommerup and Ingram, 1971). These symptoms obstruct nutrient and water transport, stunt the growth of the plant and consequently reduce crop yield and quality. In root galls, different life cycle stages of *P. brassicae* occur simultaneously.

Transcriptomics studies deciphered in part the mechanisms of the host – *P. brassicae* interaction in simplified experimental conditions, but not in complex soil. During both the spore germination and the primary zoospore stages, the pathogen showed high active metabolisms of chitinous cell wall digestion, starch, citrate cycle, pentose phosphate pathway, pyruvate, trehalose, carbohydrates and lipids (Schwelm *et al*., 2015a; Schwelm *et al*., 2015b; Bi *et al*., 2016). During the second phase of infection, genes involved in basal and lipid metabolism were highly expressed (Bi *et al*., 2016), as well as the *G‐protein‐coupled receptors pathway‐related* genes (Bi *et al*., 2019). These active metabolic pathways allow *P. brassicae* to take up nutrients from the host cells (Kageyama and Asano, 2009; Perez‐Lopez *et al*., 2018). During the formation of primary and secondary plasmodia, it is expected that *P. brassicae* secrets an array of effector proteins triggering growth, expansion and differentiation of infected host cells. Nevertheless, few RxLR effectors have been found in *P. brassicae* (Schwelm *et al*., 2015b; Rolfe *et al*., 2016) and no LysM‐effectors, known to interfere with chitin detection in fungal–plant interactions (Kombrink and Thomma, 2013), were detected. Some candidate potential effectors have however been identified from *P. brassicae* (Schwelm *et al*., 2015b; Rolfe *et al*., 2016; Daval *et al*., 2019), such as Crinkler (CRN)‐related proteins (Zhang *et al*., 2016), but their roles in infection and disease development have still to be identified (Perez‐Lopez *et al*., 2018). Only one effector has been characterized in detail: a predicted secreted methyltransferase that can mediate methylation of salicylic, benzoic and anthranilic acids, thereby interfering in the plant salicylic acid‐induced defence (Ludwig‐Muller *et al*., 2015).

Concerning the plant, *P. brassicae* infection altered likewise primary and secondary metabolism, as pathways involved in lipid, carbohydrate, cell wall synthesis, lignification‐related genes, arginine and proline metabolism (Ludwig‐Müller, 2008; Gravot *et al*., 2011; Gravot *et al*., 2012; Chen *et al*., 2015; Li *et al*., 2020), producing a sink of plant metabolites assimilated by the pathogen and corresponding to a metabolic cost for the infested plant. Clubroot infection also modified plant hormone homeostasis and defence responses, such as cytokinin biosynthesis, auxin homeostasis, salicylic acid and jasmonic acid metabolism (Siemens *et al*., 2006; Ludwig‐Müller, 2008; Agarwal *et al*., 2011; Schuller *et al*., 2014; Chen *et al*., 2015; Lemarie *et al*., 2015; Malinowski *et al*., 2016; Li *et al*., 2020).

During its life cycle, *P. brassicae* can establish potential relationships with microbiota from soil, rhizospheric soil and roots. Beneficial effect of various specific biocontrol microorganisms in suppressing clubroot has been demonstrated, such as *Trichoderma* spp. (Cheah *et al*., 2000), *Streptomyces* sp. (Cheah *et al*., 2001; Lee *et al*., 2008), *Heteroconium chaetospira* (Lahlali *et al*., 2014), *Streptomyces platensis* (Shakeel *et al*., 2016), *Bacillus subtilis* (Guo *et al*., 2015; Zhao *et al*., 2016), *Zhihengliuella aestuarii* B18 (Luo *et al*., 2017), *Paenibacillus kribbensis* (Xu *et al*., 2014) and *Lysobacter antibioticus* (Zhou *et al*., 2014). Most of these organisms were isolated from rhizosphere soil or root endosphere. Mechanisms by which these microorganisms protect against clubroot are not yet elucidated but could imply antifungal compounds or molecules up‐regulating host plant defence genes. In addition, the microbe abundance in *B. napus* clubroot‐infected endosphere roots was found higher in asymptomatic roots than in symptomatic roots, and the asymptomatic roots contained many microorganisms with biological control properties and plant growth promotion functions (Zhao *et al*., 2017). In Chinese cabbage, invasion by *P. brassicae* modified the rhizosphere and root‐associated community assembly during the secondary cortical infection stage of clubroot disease (Lebreton *et al*., 2019). This shows that the plant microbiota diversity can modulate the plant response to *P. brassicae* and can be considered as a potential reservoir of biocontrol microbe for clubroot prevention. Moreover, in *B. napus*, the plant–microbiota interaction has a role in plant defence against a phytophagous insect (*Delia radicum*) (Lachaise *et al*., 2017; Ourry *et al*., 2018).

In order to gain a mechanistic understanding of how soil microbes boost plant growth and defence and/or modulate the pathogen development and pathogenicity, a major challenge is then now to shift from descriptive to functional studies. The aim of this study is to understand how a single root pathogen, *P. brassicae*, interacts with its host, the oilseed rape (*B. napus*), considering the role of the soil microbial diversity as a reservoir of microbial functions related to plant resistance phenotype. To explore how the soil microbial environment may influence the functions of a pathogen and its pathogenesis, and the molecular response of the plant to the infection, we evaluated the effect of soils, obtained by an experimental approach of dilution to extinction and then harbouring different microbial diversities and functions but similar physicochemical properties, on (i) the phenotype of two plant genotypes harbouring different levels of susceptibility to the clubroot pathogen, and (ii) the transcriptomes of pathogen and host plant in interaction.

## Results

### Characterization of the microbial communities in the initial three soil conditions

The microbiological composition after recolonization of the three soils manipulated for having different microbial diversities (high diversity level [H], medium diversity level [M] or low diversity level [L]) was analysed. As expected, the three soils displayed optimal fungal and bacterial densities and similar abundances at the end of recolonization (Fig. S1). Not significant differences for the main soil physicochemical characteristics were observed between the three soils used (Table S1). The only difference concerned the nitrogen form, that was found mainly in the nitrate form in both H and M and as nitrate and ammonium in L; however, the total nitrogen amount was similar among the three soils (0.74–0.77 g kg^−1^).

**Fig. 1:**
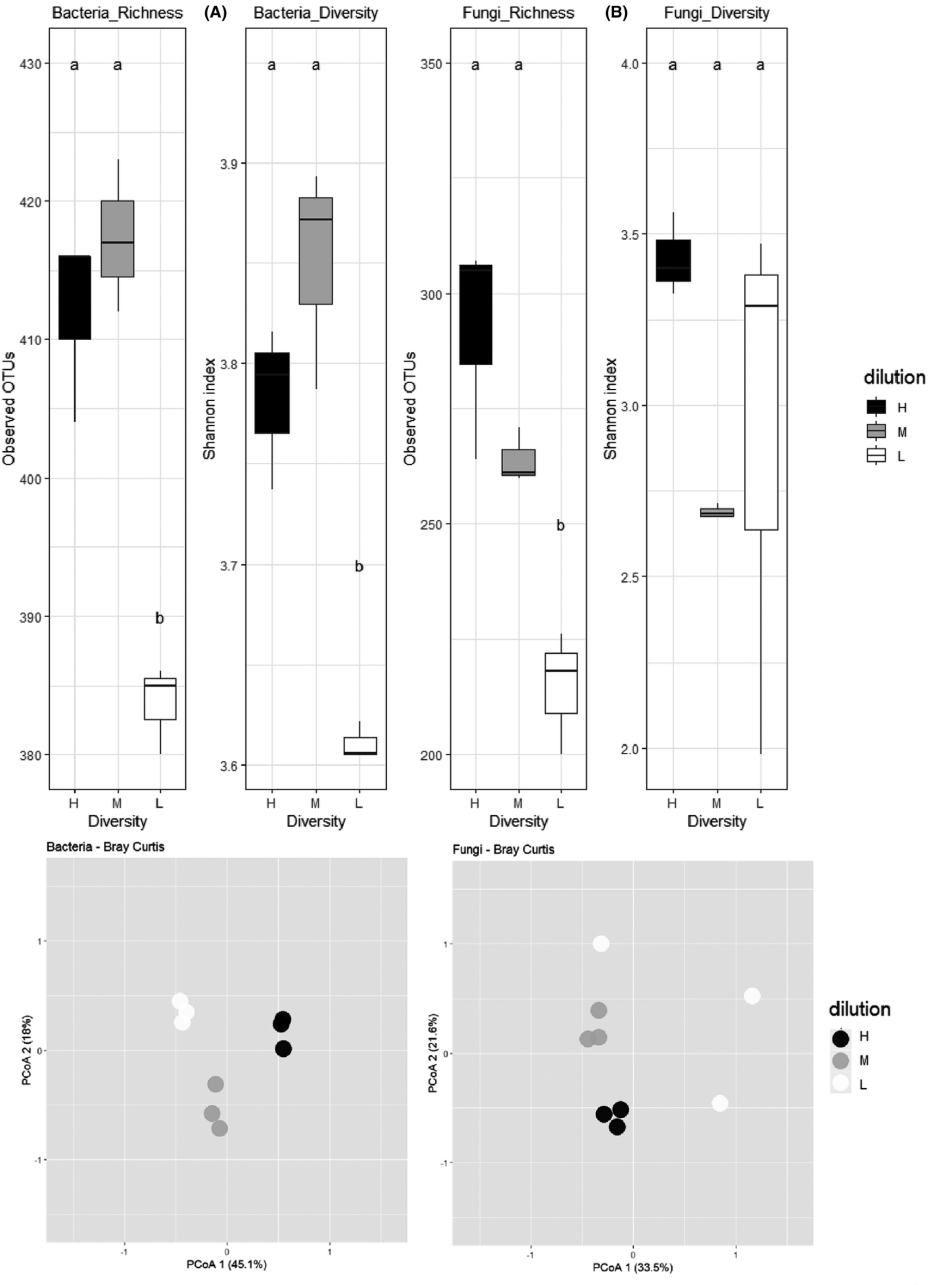
Bacterial (A) and fungal (B) richness and diversity, and communities’ structures (C) in the three soils used in this study. Mean richness (number of observed OTUs) and alpha‐diversity (Shannon index) for the three soil microbial modalities (H, high in black; M, medium in medium grey; L, low in white) are presented in bacterial (A) and fungal (B) communities. Different letters indicate statistically significant differences among communities at *P* < 0.05. Principal coordinates analysis (PCoA) projection of the communities’ structure is shown for bacteria and fungi for the H, M and L diversities (C).

We investigated the effect of the experimental dilution/recolonization on microbiota diversity. Alpha‐diversity (within each modality of soil) was analysed based on the OTUs richness and the Shannon diversity index. For bacterial kingdom (Fig. 1A), we observed a statistically significant reduction in richness and specific diversity from H or M to L microbial modalities. For fungal kingdom (Fig. 1B), the fungal richness, and to a lesser extent the fungal diversity, decreased also from H to L. Beta‐diversity (between soil modalities) was measured for the bacterial and fungal communities (Fig. 1C). The soil microbial diversities differed significantly for bacterial and fungal communities. Frequencies of bacterial and fungal phyla, genera and OTUs for each microbial modality are shown in Figure S2. At the level of phyla, both bacteria and fungi displayed similar frequencies whatever the soil modality, with *Proteobacteria* and *Ascomycota* the dominant phyla, respectively. *Bacillus* and *Pseudomonas* on one hand, and *Schizosaccharomyces* and *Fusarium* on the other hand, were major genera concerning bacteria and fungi, respectively, for the three soils.

In conclusion, the soils, obtained by microbial diversity manipulation through serial dilutions and recolonization of a single matrix, displayed similar physicochemical properties and microbial abundance, but had contrasted microbe diversity parameters affected by dilution. This experimental approach allowed us to specifically and uniquely test the effect of the microbial diversity factor on the infection of *B. napus* by *P. brassicae* and ensured that the effect of other factors related to the soil properties was not investigated.

### Modulation of the plant susceptibility to clubroot according to the soil microbiota composition

**Fig. 2:**
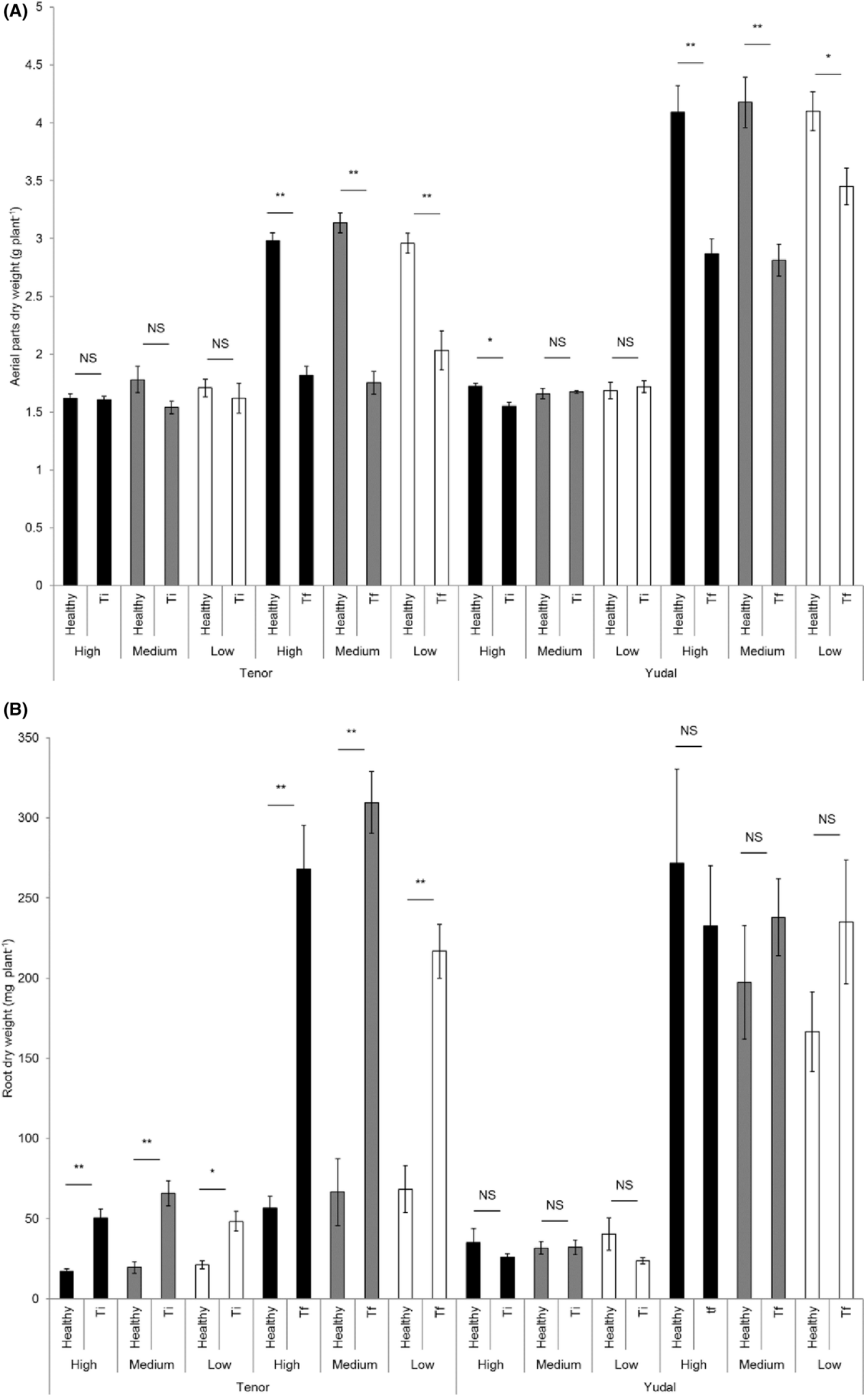
Aerial and root biomasses. The dry aerial parts (A) and roots (B) were weighted for both genotypes (Tenor and Yudal) at different days after inoculation (Ti, 28 dai; Tf 36 or 48 dai). For soil diversity, black, medium grey and white bars correspond to high (H), medium (M) and low (L) diversities, respectively. Error bars represent standard errors from the means of eight plants. **P < 0.01; *P < 0.05; NS, non‐significant.

The dry aerial parts were weighted in all experimental conditions (Fig. 2A). At Ti (intermediary time), no significant differences were measured between healthy and inoculated plants, whatever both the soil microbiota modality and the plant genotype (except a small difference in H between healthy and inoculated Yudal). On the contrary, at the final time of the experiment (Tf), the inoculated plants displayed significant reduced aerial dry weight than healthy plants, whatever both the soil microbiota modality and the host plant genotype. At this time‐point, the weight of aerial parts of both healthy and inoculated Tenor plants was weaker than in Yudal plants.

Concerning the roots (Fig. 2B), the Tenor inoculated roots showed heavier dry mass (5–6 times more) at Ti and Tf than healthy roots, for each soil microbiota modality. The Tenor healthy roots had weak growth between Ti and Tf, whatever the soil, whereas inoculated Tenor had roots 6 times heavier at Tf than at Ti. This is the result of a strong development of galls in this genotype during this period. Concerning the Yudal root dry weights, no differences between healthy and infected plants were observed whatever the microbiota soil dilution and whatever the sampling date, probably because of the small size of galls clearly visible in Yudal genotype. At Tf, Yudal healthy roots were heavier than Tenor ones because of different root developmental patterns between the two genotypes.

At each sampling time, the soil microbiota modality had overall no effect on both aerial and root dry weights of healthy and inoculated plants.

**Fig. 3:**
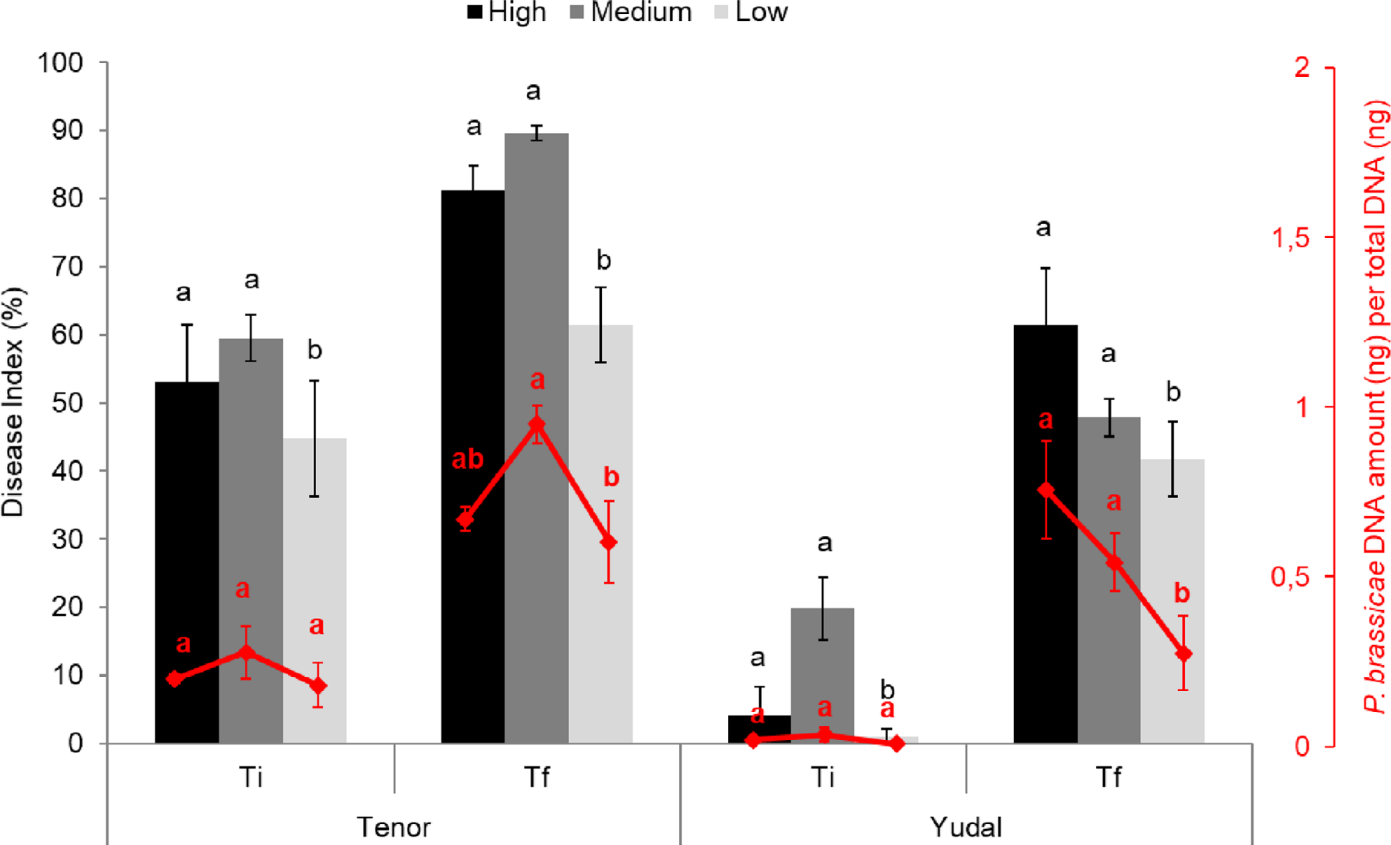
Influence of soil microbiota diversity on clubroot development. Plants were exposed to high (black), medium (grey) or low (white) soil microbial modalities during 28 (Ti), 36 or 48 (Tf) days after inoculation with the eH isolate of *P. brassicae*. The clubroot symptoms were estimated according to the disease index and the quantification of *P. brassicae* DNA by qPCR, expressed as a ratio of the 18S DNA quantity relative to the total DNA. Data are means of three biological replicates (12 plants per replicate) and error bars represent standard errors of the means. Means with different letters are statistically significantly different according to the analysis of variance test (*P* < 0.05).

At Ti and Tf, disease severity of inoculated plants was scored by determining the disease index (DI) and the DNA pathogen content (Fig. 3). For each plant genotype, the DI showed the progression of disease along time‐points: DI is about 50% at Ti and 80 % at Tf for Tenor, and less than 20% at Ti and 50% at Tf for Yudal. Whatever the soil modality and the sampling date, Yudal displayed lower DI than Tenor. This expected difference is consistent with the known level of clubroot resistance/susceptibility already described for these genotypes (Aigu *et al*., 2018). The soil microbiota modality had an effect on DI. For Tenor, at Ti and Tf, the DI was statistically significantly lower in L compared to H and M, and the highest DI was obtained in M. The DNA pathogen content followed the same pattern. At Ti, the *P. brassicae* DNA content was low, making difficult to compare the values between samples. At Tf, the DNA pathogen content was lower in L than in H and M, and higher in M, providing a bell curve. Concerning the Yudal genotype, very low DI and DNA *P. brassicae* content were observed at Ti, making difficult the interpretation of the results. At Tf, decreasing gradients of DI and pathogen DNA content were measured through soil dilutions from H to L: the less rich and diverse soil, the less plant disease and DNA pathogen content.

### Overview, mapping and validation of RNAseq data

Approximately 80–100 million (M) reads by sample were obtained, and from 86 to 93% of the reads were mapped to the reference genome that we constructed, corresponding to the *B. napus* and the *P. brassicae* concatenated genomes.

Pathogen gene expression’s profiles were clearly clustered by the host plant genotype at Ti, and both by the soil microbiota modality and the host plant genotype at Tf (Fig. S3A). No similar heatmap was performed with the *B. napus* gene expression profiles because of a huge number of expressed genes making the figure unreadable.

Hierarchical cluster analysis (HCA) (Fig. S3B) on the filtered and normalized count values concerning *P. brassicae* for each sample at Ti showed no true cluster structure in function of replicate, soil microbiota diversity or host plant genotype. On the contrary, at Tf for both host genotypes, the HCA identified separated groups for the three replicates in H, in a lesser extent in M, and a less good grouping in L. This indicated that the experimental variation was higher in the more diluted soil microbial modality (L). Concerning the *B. napus* reads, in healthy (Fig. S4A) and inoculated (Fig. S4B) plants, the analysis showed that data clustered first by the host genotype, and then by the time factor, the soil modality and the replicate.

### Modulation of the *P. brassicae* transcriptome by the soil microbiota composition

Table 1 shows the number of DEGs in *P. brassicae* and *B. napus* according to H compared to M or L, for each inoculated host genotype. The comparisons are focused on differences between modalities considered closest to the initial state of the soil (i.e. H) and the diluted conditions (M and L).

**Table 1:** Number of DEGs in *P. brassicae* and in *B. napus* depending on the soil microbiota diversity levels.

Organism in which DEGs are counted	Infection stage	Host plant genotype	H versus M		H versus L	
			Healthy plants	Infected plants	Healthy plants	Infected plants
*P. brassicae*	Ti	Yudal	nd	0	nd	0
		Tenor	nd	0	nd	1
	Tf	Yudal	nd	296	nd	0
		Tenor	nd	1827	nd	770
*B. napus*	Ti	Yudal	0	0	8	64
		Tenor	53	0	814	0
	Tf	Yudal	1852	0	3744	23
		Tenor	883	3	3945	0

DEGs, differentially expressed genes; H, high diversity modality; L, low diversity modality; M, medium diversity modality; nd, not detected; Tf, final time; Ti, intermediary time.

Concerning the *P. brassicae* transcriptome, no DEGs between the soil microbiota modalities were detected at Ti (except only one gene between H and L in infected Tenor). On the contrary, at Tf, when galls were developed, the transcriptome of *P. brassicae* was different between soils. Interestingly, *P. brassicae* displayed a higher number of DEGs when infecting Tenor (2597 DEGs between H and both M and L) than when infecting Yudal (296 DEGs).

#### Modulation of the *P. brassicae* transcriptome by the soil microbiota composition when infecting Yudal

In the interaction with Yudal, only the M condition had an effect on the *P. brassicae* gene expression compared to H at Tf (Table 1). The complete list of the DEGs is presented in the Table S2. Only nine genes among the 296 DEGs were overexpressed at M compared to H, with a small fold‐change between conditions (1.2–1.6). No particular function of these genes can be easily associated with the DI between M and H (general pathways, such as signalization and chromosome condensation). On the contrary, a higher number of *P. brassicae* genes (287) were significantly underexpressed at M compared to H, in the same way than level of disease was lower at M compared to H. We selected the top 30 most significant down‐regulated genes in M compared to H, with a fold‐change greater than 2 (Table 2). Some of these top genes are potentially involved into the transport of molecules (e.g. *FMN‐binding glutamate synthase family*, *MFS transporter Major Facilitator Superfamily*), and in development, growth and cell differentiation (e.g. *Chitin Synthase_2*, *Phosphoenolpyruvate carboxykinase*, *Glycosyltransferase*). Other genes were related to pathogenicity, including *Carbohydrate‐binding module family_18*, *Glycoside hydrolase family_16* and *NUDIX_hydrolase*.

**Table 2:** Selection of top 30 ranking *P. brassicae* highly down‐regulated genes (fold‐change> 2) in M compared to H at Tf when infecting Yudal (Y).

*P. brassicae* gene	*P. brassicae* gene expression level		Fold‐ change	Description	Enzyme codes
	In Y/H/Tf	In Y/M/Tf			
Pldbra_eH_r1s003g01588	0.43	0.02	11.42	sugar_ABC_transporter_substrate‐binding^1^	NA
Pldbra_eH_r1s004g02573	0.69	0.06	10.33	ADP‐ribosylation_factor_6^2^	NA
Pldbra_eH_r1s003g01442	0.58	0.04	8.56	calcium_calmodulin‐dependent_kinase_type_IV‐like^2^	ec:2.7.11.10
Pldbra_eH_r1s003g01889	1.85	0.34	4.76	NUDIX_hydrolase^3^	ec:3.6.1.65
Pldbra_eH_r1s008g04734	2.32	0.46	4.72	Serine_threonine‐_kinase_Sgk3^2^	ec:3.1.4.4
Pldbra_eH_r1s011g06165	4.32	0.89	4.53	UDP‐D‐xylose: L‐fucose_alpha‐1;3‐D‐xylosyltransferase_1‐like^2^	ec:2.4.1.37
Pldbra_eH_r1s015g07579	6.72	1.51	4.21	MFS_transporter^1^	NA
Pldbra_eH_r1s001g00152	1.67	0.4	4.06	carbohydrate‐binding_module_family_18^3^	ec:3.2.1.14
Pldbra_eH_r1s001g00029	7.63	1.84	4.02	WD40_repeat	NA
Pldbra_eH_r1s008g04750	5.45	1.33	3.91	methyltransferase_domain‐containing	ec:2.1.1.300
Pldbra_eH_r1s002g01072	7.08	1.78	3.89	FMN‐binding_glutamate_synthase_family^1^	ec:1.4.1.14
Pldbra_eH_r1s001g00617	8.55	2.17	3.86	glutamate_NAD(P)+^2^	ec:1.4.1.23
Pldbra_eH_r1s042g12180	3.67	0.94	3.75	calcium/calmodulin‐dependent_protein_kinase_type_IV‐like^2^	ec:2.7.11.10
Pldbra_eH_r1s007g04295	21.71	6.2	3.47	Mps1_binder^2^	NA
Pldbra_eH_r1s001g00511	40.71	11.71	3.46	serine_threonine‐_kinase_HT1^2^	ec:2.7.11.10
Pldbra_eH_r1s016g07781	26.37	7.58	3.45	chitin_synthase_2^2^	ec:2.4.1.16
Pldbra_eH_r1s034g11599	8.64	2.45	3.41	WD‐40_repeat_domain‐containing	NA
Pldbra_eH_r1s025g10321	8.55	2.46	3.38	maltose_maltodextrin_ABC_substrate_binding_periplasmic^1^	ec:2.5.1.2
Pldbra_eH_r1s014g07095	3.27	0.92	3.35	glucosamine_6‐phosphate_N‐acetyltransferase^2^	ec:2.3.1.193
Pldbra_eH_r1s001g00671	17.87	5.47	3.23	glutathione‐disulfide_reductase	ec:1.8.1.7;ec:1.8.2.3;ec:1.8.1.5
Pldbra_eH_r1s003g01550	8.87	2.75	3.15	glycosyltransferase^2^	ec:2.4.2.38
Pldbra_eH_r1s003g01890	49.53	15.74	3.13	glycoside_hydrolase_family_16^3^	ec:3.2.1.151
Pldbra_eH_r1s033g11505	14.72	4.86	3	glycosyltransferase^2^	ec:2.4.2.38
Pldbra_eH_r1s028g10813	7.61	2.63	2.8	ABC_transporter_G_family^1^	ec:3.6.1.15;ec:3.6.3.43
Pldbra_eH_r1s022g09622	47.16	17.11	2.74	phosphoenolpyruvate_carboxykinase^2^	ec:4.1.1
Pldbra_eH_r1s024g09958	32.88	12.19	2.68	phosphate_ABC_transporter_substrate‐binding^1^	ec:3.1.3.1
Pldbra_eH_r1s002g00819	6.83	2.63	2.52	cytochrome_P450	ec:1.6.2.4;ec:1.14.14.1;ec:1.14.21.7;ec:1.16.1.5;ec:1.18.1.7
Pldbra_eH_r1s028g10814	6.19	2.49	2.41	ABC_transporter^1^	ec:3.6.1.3;ec:3.6.1.15;ec:3.6.3.43
Pldbra_eH_r1s009g05056	9.63	4.23	2.28	probable_phospholipid‐transporting_ATPase_IA_isoform_X1^1^	ec:3.6.1
Pldbra_eH_r1s003g01928	56.23	27.59	2.03	chitin_synthase_2^2^	ec:2.4.1.16

Genes potentially involved in transport of molecules^1^, development and growth^2^ or pathogenicity^3^.

#### Modulation of the *P. brassicae* transcriptome by the soil microbiota composition when infecting Tenor

In the interaction with Tenor, 1827 genes of *P. brassicae* were differentially expressed at Tf between M and H (Table 1), most of them (1360 genes i.e. 75%) being overexpressed in M, and a smaller part (467 genes) underexpressed in M (Table S3A). Between L and H, there were 770 DEGs (Table S3B), with 532 (i.e. 70%) genes overexpressed in L compared to H and 238 underexpressed. In total, compared to the normal H level diversity, 621 *P. brassicae* genes were modulated both by M (out of 1827 genes, i.e. 34%) and L (out of 770 genes, i.e. 81%) conditions (Table S3C). Most of the genes regulated in L were also regulated in M. Moreover, these 621 genes displayed similar expression profiles: 450 genes were overexpressed at both M and L compared to H, and conversely for 171 genes. For these 171 genes, the fold‐change was very small (< 1.5 for 169 genes whatever the comparison between soil microbiota diversities), but the gene expression levels were elevated. On the contrary, among the 450 genes overexpressed in M or L compared to H, 346 displayed a fold‐change sharply higher than 2. The Table 3 shows the top 50 ranking by fold‐change genes among these 346 *P. brassicae* genes overexpressed in M and L compared to H. Many of them were related to functions of transport (*phospholipid‐transporting ATPase*, *FMN‐binding_glutamate synthase*, *Ammonium transporter*, *Phosphate ABC_transporter* or *Potassium transporter*), growth (*Chitin synthase_2*), detoxification (*Glutathione_S transferase*, *Zinc_C2H2_type_family*) or potential pathogenicity (*E3‐Ubiquitin ligase*, *alkaline ceramidase*, *cytosolic carboxypeptidase_4*, *serine carboxypeptidase_CPVL*).

**Table 3:** Selection of top 50 *P. brassicae* genes significantly differentially overexpressed in both M and L compared to H at Tf when infecting Tenor (T).

*P. brassicae* gene	*P. brassicae* gene expression level			Fold change T/ H versus T/ M	Fold change T/ H versus T/ L	Description	Enzyme codes
	In T/ H/ Tf	In T/ M/ Tf	In T/ L/ Tf				
Pldbra_eH_r1s023g09907	0.05	0.98	0.54	15.53	8.59	E3_ubiquitin‐_ligase_NRDP1^3^	NA
Pldbra_eH_r1s007g03979	0.1	0.6	0.66	5.3	5.8	Dynein_light_chain_Tctex‐type	NA
Pldbra_eH_r1s028g10892	0.28	1.31	1.46	4.69	5.31	Glucokinase^2^	ec:2.7.1.2, ec:2.7.1.1
Pldbra_eH_r1s035g11711	3.74	14.19	11.63	3.8	3.12	Probable_phospholipid‐transporting_ATPase_7_isoform_X1^1^	ec:3.6.1, ec:3.6.3.1
Pldbra_eH_r1s014g07222	4.08	15.48	12.7	3.75	3.08	Serine_threonine_kinase ^2^	ec:2.7.11.10
Pldbra_eH_r1s001g00753	9.55	33.81	25.43	3.53	2.66	Gamma‐glutamylcyclotransferase	ec:4.3.2.6
Pldbra_eH_r1s032g11432	2.17	7.53	6.17	3.51	2.87	Glutathione_S‐transferase	ec:1.8.1.8, ec:1.5.4.1
Pldbra_eH_r1s008g04734	0.86	3.02	2.83	3.47	3.26	Serine_threonine‐_kinase_Sgk3^2^	ec:3.1.4.4
Pldbra_eH_r1s002g01071	4.07	13.84	14.55	3.4	3.57	FMN‐binding_glutamate_synthase_family^1^	ec:1.4.1.14
Pldbra_eH_r1s002g01072	2.67	9.15	11.02	3.39	4.08	FMN‐binding_glutamate_synthase_family^1^	ec:1.4.1.14
Pldbra_eH_r1s008g04744	0.88	3.02	3.82	3.38	4.27	Alkaline_ceramidase^3^	ec:3.5.1.23
Pldbra_eH_r1s007g04295	10.89	36.75	38.11	3.37	3.5	Mps1_binder^2^	NA
Pldbra_eH_r1s002g00819	3.16	10.48	9.05	3.3	2.86	Cytochrome_P450	ec:1.14.14, ec:1.16.1.5
Pldbra_eH_r1s015g07621	9.57	31.38	28.26	3.28	2.95	Ammonium_transporter^1^	NA
Pldbra_eH_r1s008g04794	1.44	4.67	4.89	3.19	3.35	Zinc_C2H2_type_family	NA
Pldbra_eH_r1s004g02345	15.37	48.78	39.96	3.17	2.6	Cytosolic_carboxypeptidase_4^3^	ec:3.4.17, ec:3.4.19.11
Pldbra_eH_r1s027g10543	1.92	6.02	5.78	3.13	3	Probable_serine_carboxypeptidase_CPVL^3^	ec:3.4., ec:2.3.1.92
Pldbra_eH_r1s017g08171	3.41	10.66	11.56	3.12	3.39	E3_ubiquitin‐_ligase_UNKL_isoform_X1^3^	NA
Pldbra_eH_r1s001g00671	7.57	23.37	22.03	3.08	2.91	Glutathione‐disulfide_reductase	ec:1.8.1, ec:1.8.2.3
Pldbra_eH_r1s001g00511	19.71	59.05	56.06	3	2.85	Serine_threonine‐_kinase_HT1^2^	ec:2.7.11.10
Pldbra_eH_r1s003g01889	1.25	3.76	4.62	2.95	3.63	NUDIX_hydrolase^3^	ec:3.6.1.65
Pldbra_eH_r1s024g09958	15.89	46.79	42	2.94	2.64	Phosphate_ABC_transporter_substrate‐binding^1^	ec:3.1.3.1
Pldbra_eH_r1s006g03794	6.54	19.26	18.82	2.92	2.85	Chitin_synthase_D^2^	ec:2.4.1.12
Pldbra_eH_r1s056g12619	3.23	9.42	9.34	2.92	2.9	Putative_WD_repeat‐containing_protein	NA
Pldbra_eH_r1s026g10483	79.6	232.56	209.79	2.92	2.63	Lysosomal_aspartic_protease	ec:3.4.23, ec:3.4.23.2
Pldbra_eH_r1s022g09656	11.68	34.05	39.09	2.91	3.34	Potassium_transporter^1^	NA
Pldbra_eH_r1s002g00884	1.35	3.87	4.21	2.89	3.15	Glutathione_S‐transferase_kappa_1	ec:2.5.1.18, ec:1.8.1.8
Pldbra_eH_r1s015g07579	3.8	10.98	10.71	2.88	2.81	MFS_transporter^1^	NA
Pldbra_eH_r1s016g07943	1.28	3.7	4.41	2.88	3.44	Dynein_light_chain	
Pldbra_eH_r1s010g05501	5.21	15.04	17.05	2.87	3.26	WD_repeat‐containing_54_isoform_X1	NA
Pldbra_eH_r1s006g03824	3.52	10.19	11.22	2.87	3.16	Zinc_C2H2_type_family_(macronuclear)	NA
Pldbra_eH_r1s009g05121	4.39	12.28	11.05	2.78	2.51	Phosphatidylserine_decarboxylase_subunit_beta	ec:4.1.1.65
Pldbra_eH_r1s008g04760	0.66	1.79	1.84	2.76	2.83	Receptor‐interacting_serine‐threonine_kinase^2^	ec:2.7.1.107
Pldbra_eH_r1s037g11906	1.28	3.57	4.15	2.73	3.17	Phosphate_ABC_transporter_substrate‐binding_protein_PstS^1^	ec:3.1.3.1
Pldbra_eH_r1s003g01729	26.32	70.09	69.36	2.66	2.63	Chitin_synthase_(Chitin‐UDP‐_ac‐transferase)^2^	ec:2.4.1.16, ec:2.4.1
Pldbra_eH_r1s007g04126	43.01	113.52	121.98	2.64	2.84	P‐type_atpase	ec:3.6.3.7, ec:3.1.3.96
Pldbra_eH_r1s004g02678	9.23	22.88	23.24	2.48	2.52	MFS_general_substrate_transporter^1^	NA
Pldbra_eH_r1s003g01750	4.15	9.82	8.35	2.37	2.01	Phosphatidylinositol_4‐kinase_alpha^2^	ec:2.7.11.1
Pldbra_eH_r1s006g03626	7.07	16.68	20.06	2.36	2.83	Mitogen‐activated_kinase_kinase_6_isoform_X2^3^	ec:2.7.11.10
Pldbra_eH_r1s002g01126	14.32	33.48	33.58	2.34	2.34	Serine_threonine_kinase^2^	ec:2.7.11.10, ec:2.7.10.2
Pldbra_eH_r1s003g01487	3.87	9.07	10	2.33	2.57	Calcium_calmodulin‐dependent_kinase_type_1D‐like^2^	ec:2.7.11.10
Pldbra_eH_r1s007g04189	40.96	88.4	101.24	2.16	2.47	Phospholipid‐transporting_ATPase_3_isoform_X1^1^	ec:3.6.1
Pldbra_eH_r1s029g11029	3.14	1.6	1.31	1.99	2.42	TKL_kinase	NA
Pldbra_eH_r1s009g05056	8.47	16.53	19.4	1.94	2.28	Probable_phospholipid‐transporting_ATPase_IA_isoform_X1^1^	ec:3.6.1
Pldbra_eH_r1s010g05586	8.16	15.82	15.52	1.94	1.91	WD_repeat‐containing_17	NA
Pldbra_eH_r1s024g09957	26	50.22	48.97	1.93	1.88	Phosphate_ABC_transporter_substrate‐binding^1^	ec:3.1.3.1
Pldbra_eH_r1s003g01928	44.45	84.65	77.2	1.9	1.74	Chitin_synthase_2^2^	ec:2.4.1.16, ec:2.4.1
Pldbra_eH_r1s009g05057	20.38	38.26	48.44	1.88	2.37	Probable_phospholipid‐transporting_ATPase^1^	ec:3.6.1, ec:3.1.3.96
Pldbra_eH_r1s027g10545	25.49	46.93	45.97	1.84	1.8	Probable_serine_carboxypeptidase_CPVL^3^	ec:3.4.21, ec:3.4.16
Pldbra_eH_r1s001g00135	29.17	51.86	56.72	1.78	1.95	Phospholipid_transporter^1^	ec:3.6.1

Genes potentially involved in transport of molecules^1^, development and growth^2^ or pathogenicity^3^.

#### Focus on modulation of the *P. brassicae* transcriptome by the soil microbiota composition between H and M

We focused on the analyses of the *P. brassicae* gene expression between M and H at Tf because in these two soil microbiota modalities, we observed (i) the most important differences in pathogen gene expression for both plant genotypes, and (ii) a contrasted disease phenotype in function of the host plant genotype (Fig. 3): lower disease level in M versus H in Yudal and higher disease level in M versus H in Tenor.

**Fig. 4:**
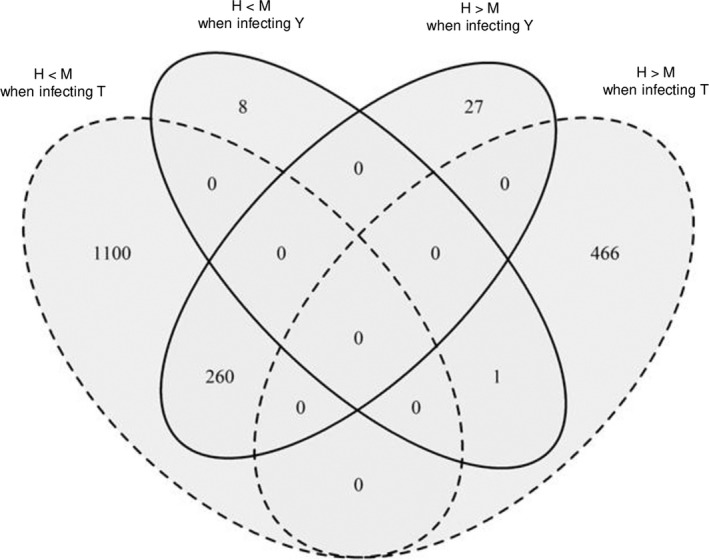
Number of *P. brassicae* differentially expressed genes (DEGs) at Tf between high (H) and medium (M) soil microbial diversity levels when infected Yudal or Tenor. The Venn diagram shows the number of significantly *P. brassicae* DEGs (*P* < 0.05) that are overexpressed (M> H) or underexpressed (M < H) in M compared to H according to the host *B. napus* genotypes (Yudal, Y; Tenor, T) at the sampling date Tf.

The sense of over‐ or underexpression profiles depending on the soil condition (H or M) was studied in detail in function of the host genotype. As shown in the Venn diagram (Fig. 4), 1360 *P. brassicae* genes (out of 1827, i.e. 74%) when infecting Tenor, and only 9 *P. brassicae* genes (out of 296, i.e. 3%) when infecting Yudal were overexpressed in M compared to H. On the contrary, almost all the genes that were regulated by the soil microbiota diversity when Yudal was infected (260 out of 296) were underexpressed in M compared to H, although they were overexpressed in M versus H when infecting Tenor. The complete list of these 260 genes with the particular expression profile depending on the H/ M levels and the host plant genotypes is indicated in the Table S4. Among these 260 genes, a selection of the top 40 genes ranked according to the fold‐change (Table 4) showed that the main functions encoded by these genes were related to the transport of molecules, the growth and development, the detoxification process and the pathogenicity. Concerning the 1100 genes specifically overexpressed in the Tenor genotype in L compared to H, most of them were related to transport of molecules (data not shown).

**Table 4:** Selection of top 40 *P. brassicae* differentially expressed genes between H and M at Tf in an opposite sense when infecting Yudal (Y) or Tenor (T)

*P. brassicae* gene	*P. brassicae gene* expression level		Fold change Y/H versus Y/M	*P. brassicae* gene expression level		Fold change T/H versus T/M	Description
	In Y/H/Tf	In Y/M/Tf		In T/H/Tf	In T/M/Tf		
Pldbra_eH_r1s001g00029	7.63	1.84	4.02	4.46	12.03	2.7	WD40_repeat
Pldbra_eH_r1s001g00152	1.67	0.4	4.06	1.16	2.79	2.38	carbohydrate‐binding_module_family_18^3^
Pldbra_eH_r1s001g00179	6.45	2.15	2.9	2.86	9.67	3.35	adenylate_guanylate_cyclase^3^
Pldbra_eH_r1s001g00511	40.71	11.71	3.46	19.71	59.05	3	Serine_threonine‐_kinase_HT1^2^
Pldbra_eH_r1s001g00617	8.55	2.17	3.86	4.59	11.96	2.6	glutamate_NAD(P)+^2^
Pldbra_eH_r1s001g00671	17.87	5.47	3.23	7.57	23.37	3.08	glutathione‐disulfide_reductase^4^
Pldbra_eH_r1s001g00753	42.72	16.1	2.64	9.55	33.81	3.53	gamma‐glutamylcyclotransferase^4^
Pldbra_eH_r1s002g00819	6.83	2.63	2.52	3.16	10.48	3.3	cytochrome_P450^4^
Pldbra_eH_r1s002g00884	1.82	0.48	3.42	1.35	3.87	2.89	glutathione_S‐transferase_kappa_1_[Rhodotorula_toruloides_NP11]^4^
Pldbra_eH_r1s002g01072	7.08	1.78	3.89	2.67	9.15	3.39	FMN‐binding_glutamate_synthase_family^1^
Pldbra_eH_r1s003g01442	0.58	0.04	8.56	0.29	1.19	4.01	calcium_calmodulin‐dependent_kinase_type_IV‐like^2^
Pldbra_eH_r1s003g01550	8.87	2.75	3.15	6.1	16.04	2.62	Glycosyltransferase_uncharacterized^2^
Pldbra_eH_r1s003g01889	1.85	0.34	4.76	1.25	3.76	2.95	NUDIX_hydrolase^3^
Pldbra_eH_r1s003g01890	49.53	15.74	3.13	26.18	68.19	2.6	glycoside_hydrolase_family_16^3^
Pldbra_eH_r1s003g01928	56.23	27.59	2.03	44.45	84.65	1.9	chitin_synthase_2^2^
Pldbra_eH_r1s006g03824	6.92	2.18	3.1	3.52	10.19	2.87	Zinc_C2H2_type_family_(macronuclear)^4^
Pldbra_eH_r1s007g04126	75.85	26.51	2.85	43.01	113.52	2.64	p‐type_atpase
Pldbra_eH_r1s007g04295	21.71	6.2	3.47	10.89	36.75	3.37	Mps1_binder^2^
Pldbra_eH_r1s008g04734	2.32	0.46	4.72	0.86	3.02	3.47	Serine_threonine‐_kinase_Sgk3^2^
Pldbra_eH_r1s008g04750	5.45	1.33	3.91	3.65	10.15	2.78	methyltransferase_domain‐containing
Pldbra_eH_r1s008g04794	3.37	0.84	3.69	1.44	4.67	3.19	zinc_C2H2_type_family^4^
Pldbra_eH_r1s009g05056	9.63	4.23	2.28	8.47	16.53	1.94	probable_phospholipid‐transporting_ATPase_IA_isoform_X1^1^
Pldbra_eH_r1s009g05121	8.82	2.71	3.2	4.39	12.28	2.78	phosphatidylserine_decarboxylase_subunit_beta
Pldbra_eH_r1s011g06165	4.32	0.89	4.53	2.28	7.67	3.34	UDP‐D‐xylose:L‐fucose_alpha‐1, 3‐D‐xylosyltransferase_1‐like^2^
Pldbra_eH_r1s014g07095	3.27	0.92	3.35	1.72	5.57	3.17	glucosamine_6‐phosphate_N‐acetyltransferase^2^
Pldbra_eH_r1s015g07579	6.72	1.51	4.21	3.8	10.98	2.88	MFS_transporter^1^
Pldbra_eH_r1s016g07781	26.37	7.58	3.45	13.19	38.91	2.95	chitin_synthase_2^2^
Pldbra_eH_r1s022g09622	47.16	17.11	2.74	22.8	62.09	2.72	phosphoenolpyruvate_carboxykinase^2^
Pldbra_eH_r1s022g09656	23.28	7.19	3.21	11.68	34.05	2.91	potassium_transporter^1^
Pldbra_eH_r1s024g09958	32.88	12.19	2.68	15.89	46.79	2.94	phosphate_ABC_transporter_substrate‐binding^1^
Pldbra_eH_r1s025g10321	8.55	2.46	3.38	4.5	10.67	2.38	Maltose_maltodextrin_ABC_substrate_binding_periplasmic^1^
Pldbra_eH_r1s027g10543	2.67	0.79	2.96	1.92	6.02	3.13	probable_serine_carboxypeptidase_CPVL^3^
Pldbra_eH_r1s028g10813	7.61	2.63	2.8	2.22	7.21	3.23	ABC_transporter_G_family^1^
Pldbra_eH_r1s028g10814	6.19	2.49	2.41	1.74	6.07	3.42	ABC_transporter^1^
Pldbra_eH_r1s033g11505	14.72	4.86	3	10.02	24.24	2.42	Glycosyltransferase_uncharacterized^2^
Pldbra_eH_r1s034g11599	8.64	2.45	3.41	3.73	11.8	3.15	WD‐40_repeat_domain‐containing
Pldbra_eH_r1s035g11711	7.18	2.74	2.57	3.74	14.19	3.8	probable_phospholipid‐transporting_ATPase_7_isoform_X1^1^
Pldbra_eH_r1s042g12180	3.67	0.94	3.75	1.55	5.79	3.66	calcium/calmodulin‐dependent_protein_kinase_type_IV‐like^2^
Pldbra_eH_r1s056g12619	5.28	1.46	3.59	3.23	9.42	2.92	putative_WD_repeat‐containing_protein
Pldbra_eH_r1s058g12634	7.62	2	3.67	4.64	12.69	2.74	peptidase_M14

Genes potentially involved in transport of molecules^1^, development and growth^2^, pathogenicity^3^ or detoxification^4^.

#### Modulation of the *P. brassicae* transcriptome by the host plant genotype in each condition of soil microbiota composition

**Fig. 5:**
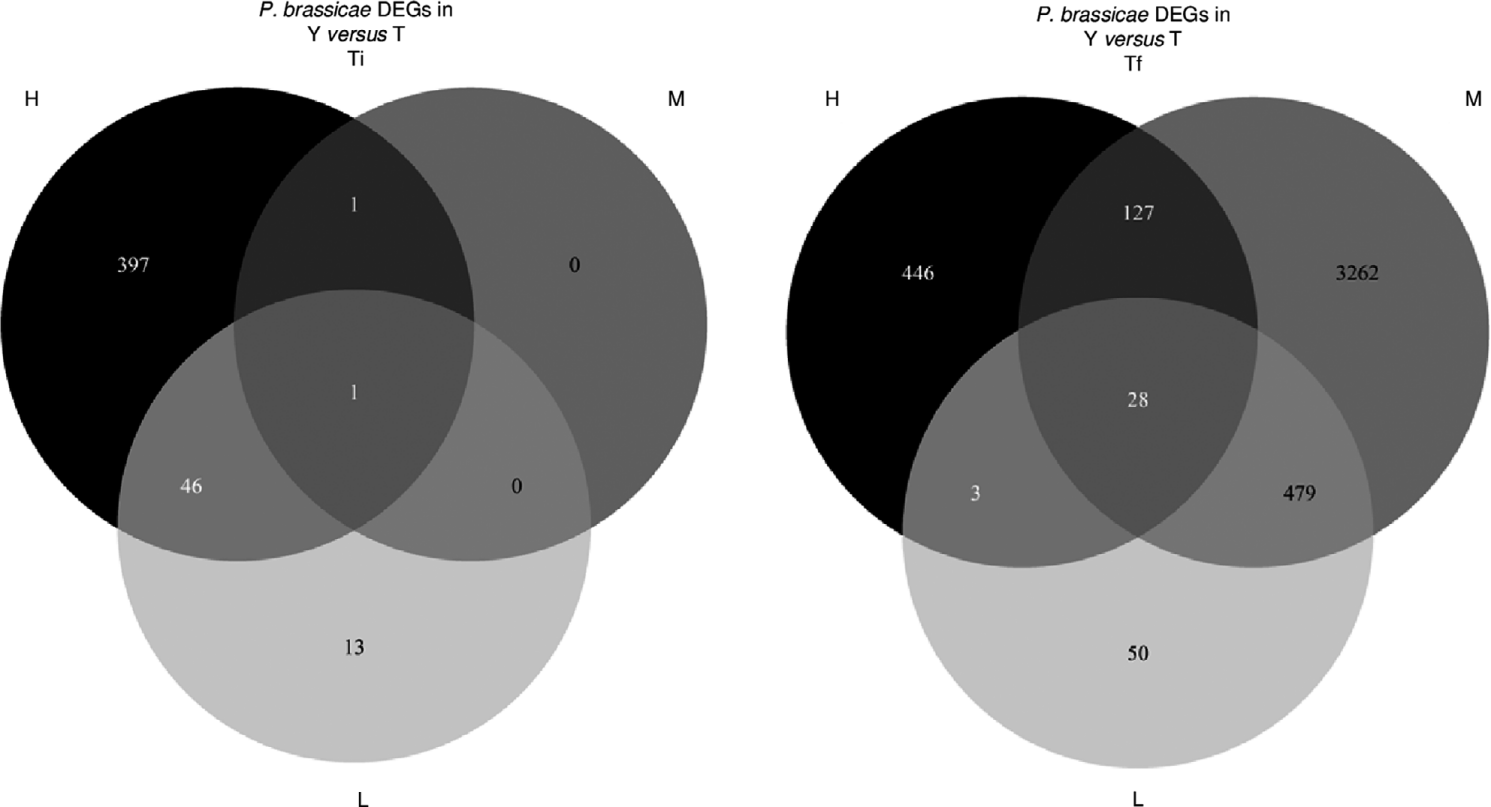
Number of *P. brassicae* differentially expressed genes (DEGs) in function of the host plant genotype for each soil microbial diversity level. The Venn diagrams show the number of significantly DEGs (*P* < 0.05) according to the host *B. napus* genotypes (T, Tenor; Y, Yudal) for each soil microbial diversity level (H, High; M, Medium; L, Low) at the sampling dates Ti and Tf.

The number of DEGs in *P. brassicae* according to the plant host genotype for each microbial diversity is presented in Figure 5. At Ti, the effect of the host plant genotype on *P. brassicae* transcriptome was more important in H (445 DEGs) than M (2 DEGs) or L (60 DEGs), and most of the DEGs in L (78%) were also DEGs in H. Only one gene (with no known annotation) was differentially expressed according to the host genotype whatever the soil microbiota diversity. At Tf, a higher number of DEGs was found between host genotypes for each diversity than at Ti. The effect of the plant genotype was around 6 times more important in M (3896 DEGs) than in H (604 DEGs) or L (560 DEGs). This is coherent with the observation that the M condition led to a contrasted disease phenotype in function of the host plant genotype (Fig. 3: higher disease level in H versus M for the infected Yudal and lower disease level in H versus M for the infected Tenor). There were only 31 common DEGs between H and L and 155 between H and M, showing a particular *P. brassicae* transcriptome in function of the plant genotype in H. On the contrary, most of the DEGs in L were also DEGs in M. Finally, 84% (3262 out of 3896) of the *P. brassicae* DEGs between host genotypes in M were specific of this soil microbiota diversity. A core of 28 DEGs was common to the three soil modalities; among them, whatever the soil microbiota diversity, 11 and 17 were under‐ or overexpressed in Tenor compared to Yudal, respectively. These genes displayed either unknown functions or functions of the general metabolism (data not shown).

### Modulation of the *B. napus* transcriptome by the soil microbiota composition

The results of soil diversity manipulation (M versus H and L versus H) at Ti and Tf on the *B. napus* transcriptome for each genotype, both in healthy and infected plants, are shown in the Table 1.

#### Modulation of the Yudal transcriptome by the soil microbiota composition

In healthy Yudal, a very moderate soil condition’s effect on DEGs number at Ti (0 to 8 genes), and a higher effect at Tf (1852 to 3744 genes) were measured.

In infected Yudal, the M condition did not modify the gene expression compared to H, although 64 genes at Ti (Table S5A) and 23 genes at Tf (Table S5B) were differentially expressed between L and H. Interestingly, the Yudal transcriptome was modified by L at Ti, although no effect of the diversity on plant disease phenotype was significantly detectable at this stage (Fig. 3). At Tf, the number of the genes that were down/up‐regulated was less than at Ti despite a more pronounced difference in disease phenotype between L and H. In Table 5, is shown a selection of *B. napus* genes for which the expression was greatly different in Yudal between L and H. The DEGs included a large number of genes encoding various proteins involved in plant defence, and particularly in hormonal pathways.

**Table 5:** Selection of top Yudal differentially expressed genes between H and L at Ti (A) and Tf (B) when infected by *P. brassicae*

(A) At Ti.					
*B. napus* gene	*B. napus* gene expression level			Fold change	Description
	In Y/H/Ti	In Y/L/Ti			
BnaC03g17080D	30.98	0.69		38.50	CYP71A13^1^
BnaA03g14120D	38.61	2.07		17.82	CYP71A13 = cytochrome P450, family 71, subfamily A, polypeptide 13^1^
BnaA09g41170D	32.90	2.73		11.44	Tyrosine aminotransferase 3^3^
BnaA01g28810D	70.33	7.29		9.42	Legume lectin family protein^1^
BnaC09g43040D	9.48	1.04		8.69	GHMP kinase family protein^2^
BnaA01g12970D	13.16	1.37		8.52	CysteineNArich RLK (RECEPTORNAlike protein kinase) 21^2^
BnaC04g45990D	182.19	22.53		8.05	Serine protease inhibitor, potato inhibitor INAtype family protein^1^
BnaC01g41330D	21.95	3.05		6.75	NucleotideNAdiphosphoNAsugar transferase^1^
BnaA09g00870D	497.35	75.10		6.61	Glutathione SNAtransferase F3^1^
BnaA04g27530D	30.36	5.01		6.05	NA
BnaC04g28910D	17.89	3.04		5.53	FAD/NAD(P)NAbinding oxidoreductase family protein^1^
BnaC02g43390D	26.72	4.70		5.51	0
BnaA04g03320D	70.82	13.00		5.46	JasmonateNAregulated gene 21^3^
BnaC01g36670D	117.35	22.88		5.09	CYP72A9^1^
BnaA05g25490D	18.82	3.61		5.04	Unknown protein
BnaA05g03980D	67.18	13.27		4.98	Beta glucosidase 27^1^
BnaC09g16910D	1658.97	355.44		4.67	GDSLNAlike Lipase/Acylhydrolase superfamily protein^1^
BnaA05g03390D	21.90	4.89		4.42	Trypsin inhibitor protein 1
BnaC03g17010D	14.16	3.13		4.35	Thioredoxin superfamily protein^1^
BnaA03g60240D	42.64	10.71		3.84	Seven transmembrane MLO family protein^2^
BnaA09g53990D	52.91	13.49		3.84	Pinoresinol reductase 1^1^
BnaA06g03570D	1.41	8.55	5.60		AuxinNAresponsive GH3 family protein^3^
BnaA03g07790D	2.43	14.87	5.58		ChaperoninNAlike RbcX protein

(B) At Tf.				
*B. napus* gene	*B. napus* gene expression level		Fold change	Description
	In Y/H/Tf	In Y/L/Tf		
BnaA03g55570D	14.21	0.00	112.52	Sulfotransferase 2A^2^
BnaC01g29150D	18.11	0.11	78.16	DefensinNAlike (DEFL) family protein^1^
BnaAnng01940D	63.38	11.11	5.52	Sulfotransferase 2A^2^
BnaA09g50540D	29.06	6.90	4.21	2NAoxoglutarate (2OG) and Fe(II)NAdependent oxygenase superfamily protein^1^
BnaA05g07580D	67.14	16.48	4.04	DonNAglucosyltransferase 1^2^
BnaAnng38720D	23.76	7.16	3.39	MATE efflux family protein^2^
BnaC02g22290D	6.75	28.87	3.75	NA
BnaC09g18860D	429.43	1547.24	3.59	Cytochrome P450, family 707, subfamily A, polypeptide 3^2^

A. Genes potentially involved in plant defence and stress response^1^, signalization pathway^2^ or hormonal and jasmonic acid pathways^3^.

B. Genes potentially involved in plant defence and stress response^1^ or hormonal and jasmonic acid pathways^2^.

#### Modulation of the Tenor transcriptome by the soil microbiota composition

In healthy Tenor, similar expression profiles to those of healthy Yudal were found, with a moderate number of DEGs at Ti between M and H (53 genes), and higher number between L and H (814 corresponding nearly to only 8 ‰ of the total number of expressed genes in *B. napus*). At Tf, 883 DEGs between M and H, and 3945 between L and H were found. In infected Tenor, no genes were differentially expressed between the soil conditions, except only 3 genes between M and H at Tf.

#### Host plant genotype’s effect on the *B. napus* transcriptome in each modality of soil microbiota composition

The global view of DEGs in healthy and infected plants of the two host genotypes, according to the soil microbiota modality and the interaction time is illustrated in Venn diagrams (Fig. S5). The number of *B. napus* DEGs between genotypes was huge in healthy and infected plants, and largely the same whatever the soil microbiota (14 789–27 537). In all the studied conditions, the effect of the genotype on plant transcriptome was very marked since about one third of the genes was differentially expressed between genotypes whatever the diversity, the time of interaction and the presence or not of the pathogen.

#### Modulation of the *B. napus* transcriptome by the infection stage in each modality of soil microbiota composition

The number of *B. napus* DEGs in each soil microbiota condition according to the infection stage showed high changes in transcript levels (Fig. S6). The high number of DEGs was retrieved for both host plant genotypes, infected or not, and for the three soil conditions. Whatever the diversity of the soil microbial community, the number of DEGs was quite similar for both genotypes in healthy plants. In infected plants, the number of DEGs in Yudal was slightly higher than in Tenor, particularly in H (19230 and 13771 DEGs in Yudal and Tenor, respectively) and L (15560 and 10547 DEGs in Yudal and Tenor, respectively). Depending on the soil condition, both genotypes displayed 25 to 50% of common DEGs set between Ti and Tf. A moderate number of DEGs was shared between plant genotypes and soil microbiota diversities (1388 and 2192 in healthy and infected plants, respectively).

By focusing more specifically on the *B. napus* genes that were differentially expressed between Ti and Tf for both infected genotypes and for the three soil’s conditions, 2192 genes were recovered (Fig. S6). Most of them were regulated in the same sense for Yudal and Tenor according to the time‐point (Fig. S7A). A slight part of genes had opposite sense of expression between plant genotypes: 6 genes were underexpressed in Yudal at Ti compared to Tf but overexpressed in Tenor at Ti compared to Tf, and 34 genes were overexpressed in Yudal at Ti compared to Tf but underexpressed in tenor at Ti compared to Tf (Fig. S7A). The annotation of 33 genes out of the 40 was retrieved (Fig. S7B). Concerning the genes overexpressed in Yudal and underexpressed in Tenor at Ti compared to Tf, they were mainly related to growth and plant development. Other genes were related to the response to disease, or involved in hormonal signalization. Two genes (*WRKY DNA binding protein 11* and *Basic region/leucine zipper motif 53*) encoding for transcription factors were also differentially expressed between Ti and Tf in a different way according to the plant genotype.

## Discussion

The plant‐associated microbiota is more and more recognized as important determinant of plant health and pathogen suppression. As main ways to control clubroot such as crop rotations and cultivation of varieties carrying major resistance genes (Dixon, 2009; Hwang *et al*., 2012) have shown their limits, there is a need to design alternative and durable methods based on ecological concepts. Exploring and understanding the mechanisms of disease regulation by microbiota could contribute to the emergence of innovative plant protection strategies.

Our research provides an extensive study of molecular mechanisms involved in complex host–pathogen interactions modulated by soil microbiota composition, using dual RNA‐seq to simultaneously capture the transcriptome of the two interacting partners. This approach has been applied to investigate a variety of host–pathogen relationships in major plant diseases in simplified *in vitro* experiments (Oh *et al*., 2008; Westermann *et al*., 2012; Wolf *et al*., 2018). Our study upgraded the dual RNA‐seq approach in more complex and realistic interaction’s conditions.

### Soil microbiota composition and clubroot phenotypes

The soil microbial diversity manipulation through serial dilutions (dilution to extinction experiment) led to a decreasing gradient of bacterial and fungal richness and a modification of community’ structure, as previously described (Lachaise *et al*., 2017), allowing experiments in controlled conditions using different microbial diversity reservoirs. Soils also had common properties overall, except for a small difference in the predominant form of nitrogen: at sowing, out of a total nitrogen content equal between the three soils, nitrogen was mainly found as nitrate form in both H and M, and ammonium in L. Different studies showed that nitrogen can have a role on soil bacterial colonization and composition. For example, high nitrogen rates increased both root exudation and the abundance of soil bacteria in maize (Zhu *et al*., 2016), and the plant responses to nitrogen availability, particularly in terms of nitrogen uptake and root exudate profiles, can act as a drivers of microbial community composition (Varanini *et al*., 2018). In our study, the all plants were watered in the same way with a nutrient solution containing nitrogen in content and form that were not limiting factors for plant growth. So a balancing in nitrogen amount can be expected between experimental conditions and thus no important differences driven by the nitrogen occurred in the plant–microbe–soil system in the microbial community structure for each soil.

We found that the microbial diversity modulated the clubroot development, in different patterns according to the host plant genotype. Interestingly, when Yudal was infected, the decrease in microbial diversity led to a proportional decrease in disease level, and in infected Tenor, a bell curve of disease level according to microbial diversity was found. The invasion of pathogens is often described as linked to the level of microbial community’s diversity and connectedness (Yan *et al*., 2017; Mallon *et al*., 2018). It is also known that rhizosphere and endophytic microbial communities, that play key roles in controlling pathogens (Erlacher *et al*., 2014; Podolich *et al*., 2014; Lugtenberg *et al*., 2016; Hassani *et al*., 2018), are recruited from the communities of microorganisms in the soil in part in a plant‐specific controlled way. It is indeed proved that different genotypes of the same plant species may have significant impacts on selecting rhizospheric partners through production of diverse root exudates (Bulgarelli *et al*., 2012; Mahoney *et al*., 2017). For instance, root‐associated microbiota displaying reproducible plant genotype associations was recently identified in maize (Walters *et al*., 2018). Genotype effects of the plant hosts can be also more important for individual microbial species (Haney *et al*., 2015). The difference in modulation of clubroot by the soil microbial diversity between Yudal and Tenor, as well as the higher changes in *P. brassicae* transcript levels in function of soil microbiota composition when Tenor was infected compared to Yudal, could be due to a plant genotype’s effect on the process of microbial recruitment. More particularly, missing microbes, or prevalence of ‘helper’ microbes, or changes in the strength and connection of the microbes’ network between H, M or L conditions can support the disease’s outbreak (Blaser, 2014). Moreover, we previously showed that not only the structure of microbial communities associated with the rhizosphere and roots of healthy Brassica plants (*B. rapa*) evolved over time, but also that the invasion by *P. brassicae* changed root and rhizosphere microbial communities already assembled from the soil (Lebreton *et al*., 2019). All these results highlighted the complexity of the microbial interactions in soil, including interactions between microorganisms, between microbes and plant, and between microbes and pathogen.

### Soil microbiota composition and *P. brassicae* transcriptome

The global view of distribution of DEGs according to the soil microbiota composition, in each plant genotype and time‐point, showed that the *P. brassicae* transcriptome was not only more modulated when infected Tenor than Yudal, but also most strongly activated at Tf than Ti. During its life cycle, *P. brassicae* survives in soil in the form of resting spores. Sensing signal molecules, such as host root exudate production or specific soil environment, is essential to exit dormancy, trigger germination and begin the initial step of the life cycle inside the root: at this stage, suitable conditions in environment, such as the soil microbial diversity and composition, are necessary. Bi *et al*. (2019) showed that *P. brassicae* is able to have perception of external signals thanks to specific signalling pathway and to adapt to its environment. In our study, the very early step of interaction between *P. brassicae* spores and soil microbiota was not measured. But the higher *P. brassicae* transcriptome modulation at Tf than at Ti highlighted the secondary cortical infection stage of clubroot disease as crucial for interaction between *P. brassicae* and the microbiota. In the same way, the root and rhizosphere‐associated community assemblies in *B. rapa*, particularly the endophytic bacterial communities, were also strongly modified by *P*. *brassicae* infection during this stage (Lebreton *et al*., 2019). Thus, the disturbance consequences of the interactions between *P*. *brassicae* and the endophytic communities inside the roots occurred at the tardive date of sampling, and the effect of soil environment on *P. brassicae* transcriptome was thereby measurable at the stages where the pathogen was in a close interaction with its host.

#### The soil microbiota composition affects the expression of *P. brassicae* genes potentially involved in the transport of molecules

At Tf, higher *P. brassicae* amount (and DI) were found in H compared to M in infected Yudal, whereas lower in H compared to M when infected Tenor. The DEGs in this same sense as *P. brassicae* amount between H and M were particularly analysed for both infected host plant genotypes (Tables 2, 3, 4), and studied for their potential involvement in different functions. This is for example the case for several genes, overexpressed in conditions where DNA *P. brassicae* content was higher, that were related to functions of molecule transport. The loss of key biosynthetic pathways is indeed a common feature of parasitic protists, making them heavily dependent on scavenging nutrients from their hosts. Salvage of nutrients by parasitic protists is often mediated by specialized transporter proteins that ensure the nutritional requirements. This is the case of genes coding for a FMN‐binding_glutamate_synthase, a complex iron–sulphur flavoprotein that plays a key role in the ammonia assimilation pathways also found in bacteria, fungi and plants (van den Heuvel *et al*., 2004; Gaufichon *et al*., 2016), and for a phospholipid‐transporting ATPase, a Phosphate_ABC_transporter or a potassium transporter. Some transporters, such as the Ammonium_transporters are also expressed during host colonization and pathogenicity in fungus because of the importance of ammonia in host alkalinization (Shnaiderman *et al*., 2013; Vylkova, 2017). The soil microbiota composition and the subsequent recruitment of endophyte microbes by the plant could affect the *P. brassicae* ability to recruit nutriments from the host because of potential competition for resource (Bauer *et al*., 2018).

#### The soil microbiota composition affects the expression of *P. brassicae* genes potentially involved in growth and development

Other examples of DEGs between soil microbial diversities with expression profiles correlated to clubroot development were related to functions of growth, development and cell differentiation. For instance, the gene coding for a chitin synthase, essential for the cell wall chitin depositions during resting spore maturation, was overexpressed in conditions where clubroot symptoms were more pronounced. The chitin‐related enzymes are enriched in *P. brassicae* genome (Schwelm *et al*., 2015b; Rolfe *et al*., 2016; Daval *et al*., 2019). Deletion of *chitin synthase* genes in fungi most often results in developmental defects, which include defective infection structure development or defunct invasive growth (Kong *et al*., 2012; Liu *et al*., 2016). Concerning the gene coding for a Phosphoenolpyruvate_carboxykinase, its differential expression could make possible to *P. brassicae* a glucose‐independent growth (Nitzsche *et al*., 2017). The differential expression of a gene coding for a glycosyltransferase could facilitate the growth as shown in filamentous pathogenic fungi (King *et al*., 2017).

#### The soil microbiota composition affects the expression of *P. brassicae* genes potentially involved in pathogenicity

Some *P. brassicae* genes coding for potential pathogenicity factors, that were overexpressed in M compared to H in Tenor and/or underexpressed in M compared to H in Yudal, may explain in part the different disease phenotype observed in function of the soil microbial diversities’ conditions.

This was the case for the gene encoding a glutathione transferase that was overexpressed in conditions of important clubroot development symptoms. Glutathione transferases represent an extended family of multifunctional proteins involved in detoxification processes and tolerance to oxidative stress. In *Alternaria brassicicola*, glutathione transferases participate in cell tolerance to isothiocyanates, allowing the development of symptoms on host plant tissues (Calmes *et al*., 2015). The pathogenicity of *P. brassicae* could be partly related to its ability to protect itself against such plant defences compounds.

For other genes putatively related to pathogenicity, we found the same trend of overexpression in conditions of important clubroot development. The E3‐Ubiquitin ligase is described as a microbial effector protein that evolved the ability to interfere with the host E3‐Ub‐ligase proteins to promote disease (Duplan and Rivas, 2014) and functional characterization was recently described (Yu *et al*., 2019). The alkaline ceramidase is involved in the virulence of microbes like *Pseudomonas aeruginosa* (Heung *et al*., 2006). The cytosolic carboxypeptidase_4 and the serine carboxypeptidase_CPVL are also described as potential factors of virulence with a role in adherence process, penetration of tissues, and interactions with the immune system of the infected host (Monod *et al*., 2002; Muszewska *et al*., 2017). The genes coding for the Carbohydrate‐binding module_family_18 or the Glycoside_hydrolase family_16 can protect some fungi against plant defence mechanisms (Abramyan and Stajich, 2012; Liu and Stajich, 2015). For instance, CBM18‐domain proteins protect from breakdown by chitinase in some fungi (Liu *et al*., 2016). In Plasmodiophorids, proteins containing a CBM18 domain could bind to the chitin in order to promote modification into chitosan, a weaker inducer of immune responses than chitin in many plants (Schwelm *et al*., 2015b).

Finally, a conserved effector gene in the genomes of a broad range of phytopathogenic organisms across kingdoms (bacteria, oomycetes, fungi) (Dong and Wang, 2016; Singh *et al*., 2018), the *NUDIX_hydrolase*, was found overexpressed in conditions where clubroot symptoms were highest, according to the soil microbial diversity. In *Arabidopsis thaliana* infected by *P. brassicae*, proteomics studies had already detected an upregulation of the NUDIX protein (Devos *et al*., 2006). NUDIX effectors have been validated as pathogenesis players in a few host–pathogen systems, but their biological functions remain unclear (Dong and Wang, 2016). Further studies are necessary to decipher if *P. brassicae* might share strategy involving NUDIX effectors described in other plant pathogens. The *NUDIX* gene is a good pathogenicity candidate gene, potentially responsible for *P. brassicae* infection and subsequent disease progression and that needs to be functionally assessed.

### Soil microbiota composition and *B. napus* transcriptome

#### The host plant genotype and the infection’s kinetic strongly affect the plant transcriptome whatever the soil microbiota composition

In both healthy and infected plants, the number of *B. napus* DEGs between genotypes was huge and largely shared between soil microbiota, and the number of plants DEGs between Ti and Tf was also high for each genotype whatever the soil microbiota composition. This demonstrates that the genetic control of the developmental process is highly dynamic and complex and remains largely unknown.

The list of common DEGs between Ti and Tf in both genotypes and the three H, M, L conditions (Fig. S7) was studied more in detail, and particularly the genes overexpressed in Yudal but underexpressed in Tenor at Ti compared to Tf. These genes were mainly related to growth and plant development: *Sterol methyltransferase 3* (Schaeffer *et al*., 2001), *C2H2like zinc finger protein* (Kielbowicz‐Matuk, 2012), *BES1/BZR1 homolog 2* (Yin *et al*., 2005), *WUSCHEL‐related homeobox 4* (Zhao *et al*., 2009), *Expansin A1* (Marowa *et al*., 2016), *Arabinogalactan protein 22* (Showalter, 2001), *Trichome BireFringence 27* (Bischoff *et al*., 2010), *SKU5 similar 17* (Sedbrook *et al*., 2002), *Transcription elongation factor (TFIIS) family protein* (Van Lijsebettens and Grasser, 2014), *Endoxyloglucan transferase A3* (Akamatsu *et al*., 1999), *KIPrelated protein 2* (Vandepoele *et al*., 2002), and *Ras‐related small GTPNAbinding family protein* (Hall, 1990). Other genes of the list were related the response to disease, like the *RING/box superfamily protein* (*family E3 ligase*) (Zeng *et al*., 2008), the *Eukaryotic aspartyl protease family protein* or the *Eukaryotic aspartyl protease family protein* (Xia *et al*., 2004), the *TRAFlike family protein* (Huang *et al*., 2016). Finally, some other genes were involved in hormonal signalization (*Auxin responsive GH3 family protein*, *Heptahelical transmembrane protein2*), in primary metabolism (*Glucose‐6‐phosphate dehydrogenase* playing a key role in regulating carbon flow through the pentose phosphate pathway), and in stress response [*Galactose oxidase/kelch repeat superfamily protein* (Song *et al*., 2013)]. Two genes encoding for transcription factors were also differentially expressed between Ti and Tf in a different way according to the plant genotype (*WRKY DNA binding protein 11* and *Basic region/leucine zipper motif 53*). The sense of expression of these genes can be correlated to the level of *P. brassicae* susceptibility of both genotypes: Yudal, known to be more resistant to clubroot than Tenor, displayed an increase of gene’s expression related to growth and disease response as potential mechanisms of resistance, whatever the microbial diversity and composition in the soil.

#### The soil microbiota composition affects the plant transcriptome

In healthy plants, the soil microbiota composition effect on plant transcriptome was similar for both genotypes: no effect at Ti and close number of DEGs at Tf. In contrast, in infected plants, only Yudal transcriptome was affected by the soil microbiota diversity, and interestingly mainly at Ti. The Yudal DEGs between L and H included a large number of genes encoding various proteins involved in plant defence, such as the CYP71A13 (phytoalexin biosynthesis), the β‐glucosidase and the nucleotide diphospho‐sugar transferase (glucosinolates’ metabolism), the Pinoresinol reductase (synthesis of lignane), the oxidoreductase family protein (terpenes’ metabolism), the lectin family protein (plant defence proteins), the serine protease inhibitor and the inhibitor INAtype family protein (antimicrobial activity), the glutathione transferase F3 (transport of defence compounds) and the Lipase/Acylhydrolase superfamily protein (growth and plant defence). These proteins may represent critical early molecules in the plant defence response before disease progression.

### Complex interactions between plant/pathogen and soil microbiota

Our study aimed to decipher the interactions between plant, pathogen and the soil microbial community to better understand the mechanisms and the host/pathogen functions involved in disease modulation. We highlighted *P. brassicae* and *B. napus* DEGs between microbial environment conditions with potential functions involved in growth and pathogenicity in the pathogen, and defence in the plant. Further studies (e.g. gene inactivation) are necessary to explore if these proteins have expected functions in the Plasmodiophorids on one hand, and in *B. napus* on the other hand.

In infected plants, even the number of DEGs remained low in *B. napus*, the expression profile was pretty opposite to that of *P. brassicae* in response to soil microbiota diversity levels:

- The plant transcriptome was more modified between H and diluted conditions for Yudal, a resistant genotype, while the pathogen transcriptome was more modified between soil microbial modalities when the host plant was Tenor, a clubroot susceptible genotype.
- The plant transcriptome was more modified at Ti than Tf by the soil microbial diversity, while the pathogen transcriptome was modulated later at Tf.

This host plant genotype‐dependent and time‐lagged response to the soil microbial composition between the plant and the pathogen transcriptomes suggest a complex regulatory scheme. The soil microbiome would modulate precociously the plant defence mechanisms in the partially resistant genotype but would have moderate or no effect in the susceptible plant, perhaps because of a too high disease level. In parallel, a direct effect of the soil microbiota composition (key‐species for instance) on the pathogen could also occur in the early stages of infection, with a late visible effect on the transcriptome of the pathogen. This highlights the importance to perform studies on very early steps of infection by *P. brassicae*. Moreover, a specific microbial recruitment from the soil diversity in function of the plant genotype could also occur with subsequent consequences on pathogen metabolism in later step of its development inside the roots in interaction with endophyte microbes. These latter, differentially recruited in function of the host plant genotype, could have different effect on pathogen gene expression during its development inside the roots. In turn, the plant would affect the pathogen transcriptome by modulating or not some genes involved in growth and pathogenicity. Mutant approaches (plant and pathogen) could validate these hypotheses.

The mechanisms within the microbial functions present in soils rather than just the species need also to be studied. The difference in clubroot observed according to both plant genotypes and soil diversity could be in part explained by the concept of functional redundancy (defined as the overlapping and equivalent contribution of multiple species to a particular function) on the one hand, and the non‐redundancy of rare soil microbes playing a key role in ecosystem on the other hand (Hol *et al*., 2015). Further thorough studies on microbial endophyte and rhizosphere species and functions present in both plant genotypes depending on microbial community composition are necessary to describe if some keystone microbial species/strains of specific bacteria and/or fungi could explain the clubroot phenotypes. This would require: (i) a more accurate taxonomic resolution and a more complete description (e.g. protist community) of the microbial soil compositions; (ii) a study of the functions expressed by microbial species, as described in some examples of molecular mechanisms leading to pathogen growth suppression on plant tissues found in the literature (Cordovez *et al*., 2015; Santhanam *et al*., 2015; Cha *et al*., 2016; Chapelle *et al*., 2016). For this, metatranscriptomics approach to analyse the microbial functions expressed in roots are in progress to better understand the complex interaction plant/ pathogen/ microbial environment.

## Experimental procedures

### Preparation of soils harbouring different microbial diversity levels

The soil preparation to obtain different microbial diversity levels was performed as described in (Lachaise *et al*., 2017). The soil was collected at the INRA experimental site La Gruche, Pacé, France, from the layer −10 to −30 cm. After homogenization, grinding, sieving and mixing with silica sand (2/3 soil, 1/3 sand), a part of the soil was gamma rays sterilized at 35 kGy and stabilized for 2 months. The unsterilized soil (100 g of dry soil) was suspended in 1 L of deionized water and used for serial dilution: undiluted (10^0^, high diversity level [H], considered as the reference), diluted at 10^−3^ (medium diversity level [M]) or 10^−6^ (low diversity level [L]). Three dilution processes were performed corresponding to 3 biological replicates. The sterilized soil (2.5 kg per bag) was inoculated with 300 ml of each dilution (H, M, L) and incubated in the dark at 18°C and 50% humidity for 49 days. Every week, microbial respiration and recolonization were facilitated when opening the bags under hood. The recolonization was followed by a microbiological count of formed cultivable colonies during the incubation period (Fig. S1). Water (100 ml) was added to 25 g of soil at each time of the recolonization process. The mixture was then 3‐to‐7‐fold serially diluted with water, depending on the sampling time. For bacterial counting, 1 ml of each serially diluted sample was poured and spreaded in Petri dishes containing Tryptic Soy Agar (TSA) and an antifungal compound (Nystatin 25 mg l^−1^) (3 plates per dilution). The plates were incubated at 27°C and were observed for the growth 1 to 2 days after spreading. For the fungal counting, similar procedure was used, with an acid Malt Agar medium containing penicillin (75 mg l^−1^) and streptomycin (150 mg l^−^), and an incubation at 20°C for 5 to 10 days (5 plates per dilution). The colony forming units (CFU) was then calculated per g of soil.

### Molecular characterization of soil bacterial and fungal communities

After recolonization and before sowing, the three microbial modalities were analysed for their physicochemical composition at the Arras soil analysis laboratory (LAS, INRA, Arras, France) (Table S1) and for their microbial diversity. The GnS‐GII protocol was used for extraction of DNA from soil samples (Plassart *et al*., 2012). Briefly, DNA was extracted from 2 g of dry soil and then purified by PVPP column and Geneclean (Lebreton *et al*., 2019). PCR amplification and sequencing were performed at the GenoScreen (Lille, France) using the Illumina MiSeq ‘paired‐end’ 2 × 250 bases (16S) for bacteria and Illumina MiSeq ‘paired‐end’ 2 × 300 bases (18S) for fungi as described previously (Lachaise *et al*., 2017; Lebreton *et al*., 2019). The protist diversity was not included in the analysis. After read assembly, sequences were processed with the GnS‐PIPE bioinformatics developed by Genosol platform (Terrat et al., 2012, 2015). By performing high‐quality sequence clustering, operational taxonomic units (OTUs) were retrieved and taxonomic assignments were performed comparing OTUs representative sequences against dedicated reference databases from SILVA (Quast *et al*., 2013). The cleaned data set is available on the European Nucleotide Archive database system under the project accession number PRJEB36457. Soil samples accession numbers range from ERR3842608 to ERR3842625 for 16S and 18S rDNA.

The alpha‐diversity of the communities was analysed. To compare bacterial or fungal composition among three soil preparations, the richness of these communities was characterized by the number of OTUs found in each soil. As metric of taxonomy diversity, the Shannon diversity index was also determined [package ‘vegan’ (Oksanen *et al*., 2019)]. Since values were conformed to normality assumptions, linear models LMM function ‘lmer’, package ‘lme4’ (Bates *et al*., 2015) were used to examine differences between soil preparation for these measures. When needed, pairwise comparisons of least squares means [package ‘lsmeans’ (Lenth, 2016)] and a false discovery rate correction of 0.05 for *P*‐values (Benjamini, 2010) were performed. In order to analyse the bacterial and fungal community structure (beta‐diversity), principal coordinate analysis (PCoA) was performed on a Bray–Curtis dissimilarity matrix, obtained from OTUs data, which were normalized using a 1‰ threshold and log2‐transformed [package ‘vegan’ (Oksanen *et al*., 2019)]. A type II permutation test was performed on the PCoA coordinates to compare the community structure of the H, M and L soils [package ‘RVAideMemoire’ (Hervé, 2019)].

### Plant material and pathogen inoculation

The oilseed rape genotypes Tenor and Yudal and the eH isolate of *P. brassicae* belonging to pathotype P1 (Some *et al*., 1996; Fahling *et al*., 2003; Daval *et al*., 2019) were used in this study. Yudal and Tenor genotypes were chosen because previous assay in our laboratory showed they display different responses to clubroot infection: Tenor was more susceptible than Yudal to eH. Both *B. napus* genotypes were grown in each of the three soils (harbouring H, M or L microbial diversities). For this, seeds of oilseed rape were sown in pots filled with 400 g of experimental soils. Pots were placed in a climatic chamber, in a randomized block design with the three modalities (H, M, L) and three replicates by dilution factor. For each oilseed rape genotype, eight plants per soil microbial modality and per replicate were used. Plants were either not inoculated (healthy plants) or inoculated with a resting spore suspension of the *P. brassicae* eH isolate. For inoculum production, clubs propagated on the universal susceptible host Chinese cabbage (*B. rapa* ssp *pekinensis* cv. Granaat) were collected, homogenized in a blender with sterile water and separated by filtration through layers of cheesecloth. The resting spores were then separated by filtration through 500, 100 and 55 µM sieves to remove plant cell debris. The spore concentration was determined with a Malassez cell and adjusted to 1 × 10^7^ spores ml^−1^. Plant inoculation was done as described in Manzanares‐Dauleux *et al*. (2000): seven‐day‐old seedlings were inoculated by pipetting 1 ml of the spore suspension at 1 × 10^7^ spores ml^−1^ to the bottom of the stem of each seedling. The plants were maintained at 22°C (day) and 19°C (night) with a 16‐h photoperiod and watered periodically from the top with a Hoagland nutritive solution to provide nutrients and to maintain a water retention capacity of 70–100%.

### Phenotyping: plant characterization and disease assessment

Roots and aerial parts were sampled at two times: 28 days after inoculation (dai) (intermediary time, Ti) for both genotypes, and 36 dai and 48 dai for Tenor and Yudal (final time, Tf), respectively. The final time was chosen to have clearly visible galls on the primary and lateral roots.

At each sampling date and for each replicate, the aerial parts of 8 plants were cut, dried and weighted. As one of the three infected replicates at the final time for Tenor in L soil displayed no clubroot symptoms in any of the 8 plants, indicating that the inoculation of these plants was not successful, this sample was removed for all the analyses. The roots were cut below the collar (in the soil depth from −1 to −6 cm), separated from soil and washed twice in sterile water by vortexing 10 s. Then, the roots were transferred in a Petri dish, cut into small pieces and frozen in liquid nitrogen then stored at −80°C. After lyophilization, the dry root biomass was measured and the powder was kept until nucleic acid extraction (DNA for pathogen quantification and RNA for RNAseq analyses).

Disease was assessed at each sampling date after inoculation with *P. brassicae*. First, clubroot symptoms were evaluated by a disease index calculated with the scale previously described by Manzanares‐Dauleux *et al*. (2000). Secondly, 1 µl of DNA extracted from root samples (see 2.5) was used for quantitative PCR on the LightCycler^®^ 480 Real‐Time PCR System (Roche) to quantify *P. brassicae* amount. For this, a portion (164 bp) of the target 18S gene was amplified with the following primers: 5ʹ‐ttgggtaatttgcgcgcctg‐3ʹ (forward) and 5ʹ‐cagcggcaggtcattcaaca‐3ʹ (reverse). Each reaction was performed in 20 µl qPCR reaction with 10 µl of SYBR Green Master Mix (Roche), 0.08 µl of each primer (100 µM) and 1 µl of total DNA as template. The PCR conditions consisted of an initial denaturation at 95°C for 5 min, followed by 45 cycles at 95°C for 10 s and 64°C for 40 s. Standard curves were constructed using serial dilutions of *P. brassicae* DNA extracted from resting spores. Quantitative results were then expressed and normalized as the part of the *P. brassicae* mean DNA content in the total root‐extracted DNA.

To compare the aerial and root biomasses between modalities, linear models were used [LMM function ‘lmer’, package ‘lme4’ (Bates *et al*., 2015)]. A Wald test (α = 5%) was applied for evaluating the soil effect in the LMM model. Least Square Means (LSMeans) were calculated using the ‘lsmeans’ function of the ‘lsmeans’ package (Lenth, 2016), and the false discovery rate correction for *P*‐values (Benjamini, 2010). Pairwise comparisons of LSMeans were performed with the Tukey test (α = 5%), using the ‘cld’ function of the ‘lsmeans’ package.

Disease data were analysed using a likelihood ratio test on a cumulative link model (CLMM; ‘clmm’ function, ‘ordinal’ package). LSMeans and pairwise comparisons of LSMeans were performed as described for biomasses’ analyses.

### Nucleic acids isolation from roots

At each time‐point, the lyophilized roots from the 8 pooled plants of each genotype and each treatment (with and without *P. brassicae*) were used for nucleic acid extraction.

DNA was extracted from 30 mg of lyophilized powder root samples with the NucleoSpin Plant II Kit (Masherey‐Nagel) following the manufacturer's instructions. After verification of the DNA quality on agarose gel and estimation of the quantity with a Nanodrop 2000 (Thermo Scientific), it was used for *P. brassicae* quantification.

Total RNA was extracted from 20 mg of lyophilized powder with the TRIzol protocol (Invitrogen). RNA purity and quality were assessed with a Bioanalyser 2100 (Agilent) and quantified with a Nanodrop (Agilent).

### Library construction and Illumina sequencing

RNA‐seq analysis was performed on RNA extracted from roots tissues of two *B. napus* genotypes infected or not with resting spores of *P. brassicae* (eH isolate) grown in the three different soils (H, M, L), for three biological replicates, at Ti and Tf.

The TruSeq Stranded mRNA Library Sample Prep Kit (Illumina) was used for library construction. Library pair‐end sequencing was conducted on an Illumina HiSeq4000 (Genoscreen, Lille, France) using 2x150 bp and resulting in 2861 paired‐end millions of reads. Briefly, the purified mRNA was fragmented and converted into double‐stranded cDNA withy random priming. Following end‐repair, indexed adapters were ligated. The cDNA fragments of ~ 350 bp were purified with AMPure beads XP and amplified by PCR to obtain the libraries for sequencing. The libraries were multiplexed (six libraries per lane) and sequenced. The cleaned data set is available on the European Nucleotide Archive database system under the project accession number PRJEB36458. Samples accession numbers range from ERR3850126 to ERR3850197.

### Mapping of sequenced reads, assessment of gene expression and identification of differentially expressed genes

The read quality was undertaken for the quality scores of Q28 and for the read length of 50 nucleotides using PrinSeq. In order to use a combined host–pathogen genome as reference for alignment, the genomes of eH *P. brassicae* (Daval *et al*., 2019) and *B. napus* (Chalhoub *et al*., 2014) were concatenated, as well as the corresponding annotation files. The high‐quality reads were aligned to the concatenated files using STAR 2.5.2a_modified. Non‐default parameters were minimum intron length 10, maximum intron length 50 000 and mean distance between paired end‐reads 50 000. For the reads which can align to multiple locations (parameters set for a maximum of 6 locations), a fraction count for multimapping reads was generated. Thanks to genome annotation files, the mapped sequencing reads were assigned to genomic features using featureCounts v1.5.0‐p1 and counted. After filtering of the read counts below the threshold value (at least 0.5 counts per million in 3 samples), the count reads were then normalized with the Trimmed Mean of M values (TMM method). Concerning the *P. brassicae* reads, as the number of reads in the libraries at Ti was much smaller than at the final time (due to the differences in the infection rate and progression of the pathogen between the sampling times), the normalization was performed for Ti separately from Tf. So, analyses of *P. brassicae* were specific of each sampling time, preventing the data comparison between the time‐points. On the contrary, for *B. napus* reads, the normalization was performed on total libraries, allowing a kinetic analysis of plant transcriptome.

Differential expression analysis was performed using the EdgeR package in R. The differentially expressed genes (DEGs) with FDR ≤ 0.05 from specific comparison lists were selected for analysis. The functional annotation of DEGs was performed with blast2go 4.1.9 software. Heatmaps were generated using the ‘heatmap3’ package and Venn diagrams using the ‘VennDiagram’ packages in R.

## Conflict of interest

The authors declare no conflicts of interest.

## Supporting information


**Fig. S1.** Microbiological follow up based on the Colony Forming Units (CFU) method during the incubation period for bacteria (A) and fungi (B). H, High diversity modality; M, Medium diversity modality; L, Low diversity modality.

**Fig. S2.** Description of the main bacterial and fungal composition in the three soils. Average relative abundance (RA ± SEM) of the most abundant bacterial phyla (A), genera (B), OTUs (C), and fungal phyla (D), genera (E), OTUs (F) are shown in High (H), Medium (M) and Low (L) soil microbial diversities. For each soil, the number of replicates is *n* = 3.

**Fig. S3.** Overview of all *P. brassicae* transcriptome samples. A. Heatmaps of *P. brassicae* gene expression based on normalized data of expression values. The heatmaps are based on total reads counts for *P. brassicae* at Ti and Tf for the 3 microbial soil diversities (H, High; M, Medium, L, Low), the two plant genotypes (T, Tenor; Y, Yudal) and correspond to the mean of the three replicates. B. Hierarchical Cluster Analysis (HCA) of the filtered and normalized counts in the dual‐RNAseq analysis. The analyses are shown for *P. brassicae* reads at Ti and Tf for the 3 soil microbial diversities (H, High; M, Medium; L, Low), the two plant genotypes (T, Tenor; Y, Yudal), and the three replicates (a, b, c).

**Fig. S4.** Overview of all *B. napus* transcriptome samples. Hierarchical Cluster Analysis (HCA) of the filtered and normalized counts in the dual‐RNAseq analysis in healthy plants (A) and infected plants (B). The analyses are shown for *B. napus* reads at Ti and Tf, for the 3 soil microbial diversities (H, High; M, Medium; L, Low), the two plant genotypes (T, Tenor; Y, Yudal), and the three replicates (a, b, c).

**Fig. S5.** Number of *B. napus d*ifferentially expressed genes (DEGs) in function of the host plant genotype for each soil microbial diversity level when not infected (A) or infected by *P. brassicae* (B). The Venn diagram shows the number of significantly DEGs (*P* < 0.05) according to the host *B. napus* genotypes (T, Tenor; Y, Yudal) infected or not, for each soil microbial diversity level (H, High; M, Medium; L, Low) at the sampling dates Ti and Tf.

**Fig. S6.** Number of *B. napus* differentially expressed genes (DEGs) in function of the interaction stage for each soil microbial diversity level. The Venn diagrams show the total number of significantly DEGs (*P* < 0.05) in the *B. napus* genotypes (T, Tenor; Y, Yudal), healthy (A) or infected by *P. brassicae* (B), at each soil microbial diversity level (H, High; M, Medium; L, Low), between Ti and Tf.

**Fig. S7.** Differentially expressed genes (DEGs) in both infected *B. napus* genotypes according to the infection’s stage whatever the soil microbial diversity. A. The Venn diagram shows the number of significantly DEGs (*P* < 0.05) common in both *B. napus* genotypes (T, Tenor; Y, Yudal), and common in the three soil microbial diversity levels (H, High; M, Medium; L, Low), which are down (<) or up (>) regulated at Ti compared to Tf. B. Heatmaps of the 40 genes surrounded by a grey circle in the figure A. The expression is based on normalized data of expression values (T, Tenor; Y, Yudal; H, M, L, High, Medium, Low soil microbial diversity levels).

**Table S1.** Main physicochemical characteristics of the three soils used in this study.

**Table S2.** Description of the *P. brassicae* genes differentially expressed between H and M at Tf when infecting Yudal (−1: genes underexpressed at H compared to M; 1: genes overexpressed at H compared to M).

**Table S3.** Description of the *P. brassicae* genes differentially expressed between the different soil microbiota diversity levels at Tf when infecting Tenor. A. Description of the *P. brassicae* genes differentially expressed between H and M at Tf when infecting Tenor (−1: genes underexpressed at H compared to M; 1: genes overexpressed at H compared to M). B. Description of the *P. brassicae* genes differentially expressed between H and L at Tf when infecting Tenor (−1: genes underexpressed at H compared to L; 1: genes overexpressed at H compared to L). C. Description of the *P. brassicae* genes differentially expressed between H and M and between H and L at Tf when infecting Tenor (−1: genes underexpressed at H compared to M or L; 1: genes overexpressed at H compared to M or L).

**Table S4.** Description of the *P. brassicae* genes differentially expressed between H and M at Tf in an opposite sense when infecting Yudal or Tenor (−1: genes underexpressed at H compared to M; 1: genes overexpressed at H compared to M).

**Table S5.** Effect of soil microbiota diversity levels on infected Yudal gene expression. A. Description of the 64 *B. napus* Yudal genes differentially expressed between H and L at Ti when infected by *P. brassicae* (−1: genes underexpressed at H compared to L; 1: genes overexpressed at H compared to L). B. Description of the 23 *B. napus* Yudal genes differentially expressed between H and L at Tf when infected by *P. brassicae* (−1: genes underexpressed at H compared to L; 1: genes overexpressed at H compared to L).

## References

[mbt213634-bib-0001] Abramyan, J. , and Stajich, J.E. (2012) Species‐specific chitin‐binding module 18 expansion in the amphibian pathogen *Batrachochytrium dendrobatidis* . MBio 3: e00150‐00112. 22718849 10.1128/mBio.00150-12 PMC3569864

[mbt213634-bib-0002] Agarwal, A. , Kaul, V. , Faggian, R. , Rookes, J.E. , Ludwig‐Müller, J. , and Cahill, D.M. (2011) Analysis of global host gene expression during the primary phase of the *Arabidopsis thaliana–Plasmodiophora brassica*e interaction. Funct Plant Biol 38: 462. 32480901 10.1071/FP11026

[mbt213634-bib-0003] Aigu, Y. , Laperche, A. , Mendes, J. , Lariagon, C. , Guichard, S. , Gravot, A. , and Manzanares‐Dauleux, M.J. (2018) Nitrogen supply exerts a major/minor switch between two QTLs controlling *Plasmodiophora brassicae* spore content in rapeseed. Plant Pathol 67: 1574–1581.

[mbt213634-bib-0004] Akamatsu, T. , Hanzawa, Y. , Ohtake, Y. , Takahashi, T. , Nishitani, K. , and Komeda, Y. (1999) Expression of endoxyloglucan transferase genes in acaulis mutants of Arabidopsis. Plant Physiol 121: 715–721. 10557219 10.1104/pp.121.3.715 PMC59433

[mbt213634-bib-0005] Badri, D.V. , Zolla, G. , Bakker, M.G. , Manter, D.K. , and Vivanco, J.M. (2013) Potential impact of soil microbiomes on the leaf metabolome and on herbivore feeding behavior. New Phytol 198: 264–273. 23347044 10.1111/nph.12124

[mbt213634-bib-0006] Bakker, P. , Pieterse, C.M.J. , de Jonge, R. , and Berendsen, R.L. (2018) The soil‐borne legacy. Cell 172: 1178–1180. 29522740 10.1016/j.cell.2018.02.024

[mbt213634-bib-0007] Barret, M. , Guimbaud, J.F. , Darrasse, A. , and Jacques, M.A. (2016) Plant microbiota affects seed transmission of phytopathogenic microorganisms. Mol Plant Pathol 17: 791–795. 26850564 10.1111/mpp.12382 PMC6638484

[mbt213634-bib-0008] Bates, D.M. , Bates, D. , Maechler, M. , Bolker, B. , and Walker, S. (2015) Fitting linear mixed‐effects models using lme4. J Stat Softw 67: 1–48.

[mbt213634-bib-0009] Bauer, M.A. , Kainz, K. , Carmona‐Gutierrez, D. , and Madeo, F. (2018) Microbial wars: competition in ecological niches and within the microbiome. Microbial Cell 5: 215–219. 29796386 10.15698/mic2018.05.628 PMC5961915

[mbt213634-bib-0010] Benjamini, Y. (2010) Discovering the false discovery rate. J R Stat Soc Ser B Stat Methodol 72: 405–416.

[mbt213634-bib-0011] Berendsen, R.L. , Pieterse, C.M. , and Bakker, P.A. (2012) The rhizosphere microbiome and plant health. Trends Plant Sci 17: 478–486. 22564542 10.1016/j.tplants.2012.04.001

[mbt213634-bib-0012] Bi, K. , He, Z. , Gao, Z. , Zhao, Y. , Fu, Y. , Cheng, J. , *et al* (2016) Integrated omics study of lipid droplets from *Plasmodiophora brassicae* . Sci Rep 6: 36965. 27874080 10.1038/srep36965 PMC5118790

[mbt213634-bib-0013] Bi, K. , Chen, T. , He, Z. , Gao, Z. , Zhao, Y. , Liu, H. , *et al* (2019) Comparative genomics reveals the unique evolutionary status of *Plasmodiophora brassicae* and the essential role of GPCR signaling pathways. Phytopathology Research 1: 12.

[mbt213634-bib-0014] Bischoff, V. , Nita, S. , Neumetzler, L. , Schindelasch, D. , Urbain, A. , Eshed, R. , *et al* (2010) *TRICHOME BIREFRINGENCE* and its homolog *AT5G01360* encode plant‐specific DUF231 proteins required for cellulose biosynthesis in Arabidopsis. Plant Physiol 153: 590–602. 20388664 10.1104/pp.110.153320 PMC2879772

[mbt213634-bib-0015] Blaser, M.J. (2014) The microbiome revolution. J Clin Invest 124: 4162–4165. 25271724 10.1172/JCI78366 PMC4191014

[mbt213634-bib-0016] Brader, G. , Compant, S. , Vescio, K. , Mitter, B. , Trognitz, F. , Ma, L.J. , and Sessitsch, A. (2017) Ecology and genomic insights into plant‐pathogenic and plant‐nonpathogenic endophytes. Annu Rev Phytopathol 55: 61–83. 28489497 10.1146/annurev-phyto-080516-035641

[mbt213634-bib-0017] Bulgarelli, D. , Rott, M. , Schlaeppi, K. , Loren, Ver , van Themaat, E. , Ahmadinejad, N. , *et al* (2012) Revealing structure and assembly cues for *Arabidopsis* root‐inhabiting bacterial microbiota. Nature 488: 91–95. 22859207 10.1038/nature11336

[mbt213634-bib-0018] Calmes, B. , Morel‐Rouhier, M. , Bataille‐Simoneau, N. , Gelhaye, E. , Guillemette, T. , and Simoneau, P. (2015) Characterization of glutathione transferases involved in the pathogenicity of *Alternaria brassicicola* . BMC Microbiol 15: 123. 26081847 10.1186/s12866-015-0462-0 PMC4470081

[mbt213634-bib-0019] Cha, J.Y. , Han, S. , Hong, H.J. , Cho, H. , Kim, D. , Kwon, Y. , *et al* (2016) Microbial and biochemical basis of a Fusarium wilt‐suppressive soil. ISME J 10: 119–129. 26057845 10.1038/ismej.2015.95 PMC4681868

[mbt213634-bib-0020] Chalhoub, B. , Denoeud, F. , Liu, S.Y. , Parkin, I.A.P. , Tang, H.B. , Wang, X.Y. , *et al* (2014) Early allopolyploid evolution in the post‐Neolithic *Brassica napus* oilseed genome. Science 345: 950–953. 25146293 10.1126/science.1253435

[mbt213634-bib-0021] Chaparro, J.M. , Badri, D.V. , and Vivanco, J.M. (2014) Rhizosphere microbiome assemblage is affected by plant development. ISME J 8: 790–803. 24196324 10.1038/ismej.2013.196 PMC3960538

[mbt213634-bib-0022] Chapelle, E. , Mendes, R. , Bakker, P.A. , and Raaijmakers, J.M. (2016) Fungal invasion of the rhizosphere microbiome. ISME J 10: 265–268. 26023875 10.1038/ismej.2015.82 PMC4681858

[mbt213634-bib-0023] Cheah, L.H. , Veerakone, S. , and Kent, G. (2000) Biological control of clubroot on cauliflower with *Trichoderma* and *Streptomyces* spp. N Z Plant Prot 53: 18–21.

[mbt213634-bib-0024] Cheah, L.H. , Kent, G. , Gowers, S. , New Zealand Plant Protection Society, I.N.C. , and New Zealand Plant Protection Society, I.N.C. (2001) Brassica crops and a *Streptomyces* sp as potential biocontrol for clubroot of Brassicas In New Zealand Plant Protection, vol. 54 Rotorua: New Zealand Plant Protection Society, pp. 80–83.

[mbt213634-bib-0025] Chen, J. , Pang, W. , Chen, B. , Zhang, C. , and Piao, Z. (2015) Transcriptome Analysis of *Brassica rapa* Near‐Isogenic Lines Carrying Clubroot‐Resistant and ‐Susceptible Alleles in Response to *Plasmodiophora brassicae* during Early Infection. Front Plant Sci 6: 1183. 26779217 10.3389/fpls.2015.01183 PMC4700149

[mbt213634-bib-0026] Cordovez, V. , Carrion, V.J. , Etalo, D.W. , Mumm, R. , Zhu, H. , van Wezel, G.P. , and Raaijmakers, J.M. (2015) Diversity and functions of volatile organic compounds produced by *Streptomyces* from a disease‐suppressive soil. Front Microbiol 6: 1081. 26500626 10.3389/fmicb.2015.01081 PMC4598592

[mbt213634-bib-0027] Daval, S. , Belcour, A. , Gazengel, K. , Legrand, L. , Gouzy, J. , Cottret, L. , *et al* (2019) Computational analysis of the *Plasmodiophora brassicae* genome: mitochondrial sequence description and metabolic pathway database design. Genomics 111: 1629–1640. 30447277 10.1016/j.ygeno.2018.11.013

[mbt213634-bib-0028] Devos, S. , Laukens, K. , Deckers, P. , Van Der Straeten, D. , Beeckman, T. , Inze, D. , *et al* (2006) A hormone and proteome approach to picturing the initial metabolic events during *Plasmodiophora brassicae* infection on *Arabidopsis* . Mol Plant Microbe Interact 19: 1431–1443. 17153927 10.1094/MPMI-19-1431

[mbt213634-bib-0029] Dixon, G.R. (2009) The occurrence and economic impact of *Plasmodiophora brassicae* and clubroot disease. J Plant Growth Regul 28: 194–202.

[mbt213634-bib-0030] Dong, S. , and Wang, Y. (2016) Nudix effectors: a common weapon in the arsenal of plant pathogens. PLoS Pathog 12: e1005704. 27737001 10.1371/journal.ppat.1005970 PMC5063578

[mbt213634-bib-0031] Duplan, V. , and Rivas, S. (2014) E3 ubiquitin‐ligases and their target proteins during the regulation of plant innate immunity. Front Plant Sci 5: 42. 24592270 10.3389/fpls.2014.00042 PMC3923142

[mbt213634-bib-0032] Erlacher, A. , Cardinale, M. , Grosch, R. , Grube, M. , and Berg, G. (2014) The impact of the pathogen *Rhizoctonia solani* and its beneficial counterpart *Bacillus amyloliquefaciens* on the indigenous lettuce microbiome. Front Microbiol 5: 175. 24795707 10.3389/fmicb.2014.00175 PMC4001036

[mbt213634-bib-0033] Fahling, M. , Graf, H. , and Siemens, J. (2003) Pathotype separation of *Plasmodiophora brassicae* by the host plant. J Phytopathol 151: 425–430.

[mbt213634-bib-0034] Gaufichon, L. , Rothstein, S.J. , and Suzuki, A. (2016) Asparagine metabolic pathways in Arabidopsis. Plant Cell Physiol 57: 675–689. 26628609 10.1093/pcp/pcv184

[mbt213634-bib-0035] Gravot, A. , Grillet, L. , Wagner, G. , Jubault, M. , Lariagon, C. , Baron, C. , *et al* (2011) Genetic and physiological analysis of the relationship between partial resistance to clubroot and tolerance to trehalose in *Arabidopsis thaliana* . New Phytol 191: 1083–1094. 21599669 10.1111/j.1469-8137.2011.03751.x

[mbt213634-bib-0036] Gravot, A. , Deleu, C. , Wagner, G. , Lariagon, C. , Lugan, R. , Todd, C. , *et al* (2012) Arginase induction represses gall development during clubroot infection in Arabidopsis. Plant Cell Physiol 53: 901–911. 22433460 10.1093/pcp/pcs037

[mbt213634-bib-0037] Guo, S. , Li, X. , He, P. , Ho, H. , Wu, Y. , and He, Y. (2015) Whole‐genome sequencing of *Bacillus subtilis* XF‐1 reveals mechanisms for biological control and multiple beneficial properties in plants. J Ind Microbiol Biotechnol 42: 925–937. 25860123 10.1007/s10295-015-1612-y

[mbt213634-bib-0038] Hacquard, S. , Spaepen, S. , Garrido‐Oter, R. , and Schulze‐Lefert, P. (2017) Interplay between innate immunity and the plant microbiota. Annu Rev Phytopathol 55: 565–589. 28645232 10.1146/annurev-phyto-080516-035623

[mbt213634-bib-0039] Hall, A. (1990) The cellular functions of small GTP‐binding proteins. Science 249: 635–640. 2116664 10.1126/science.2116664

[mbt213634-bib-0040] Haney, C.H. , Samuel, B.S. , Bush, J. , and Ausubel, F.M. (2015) Associations with rhizosphere bacteria can confer an adaptive advantage to plants. Nat Plants 1: 15051. 27019743 10.1038/nplants.2015.51 PMC4806546

[mbt213634-bib-0041] Hassani, M.A. , Duran, P. , and Hacquard, S. (2018) Microbial interactions within the plant holobiont. Microbiome 6: 58. 29587885 10.1186/s40168-018-0445-0 PMC5870681

[mbt213634-bib-0042] van der Heijden, M.G. , and Hartmann, M. (2016) Networking in the plant microbiome. PLoS Biol 14: e1002378. 26871440 10.1371/journal.pbio.1002378 PMC4752285

[mbt213634-bib-0043] Hervé, M. (2019) RVAideMemoire: Testing and Plotting Procedures for Biostatistics. R package version 0.9‐73. URL https://CRAN.R‐project.org/package=RVAideMemoire.

[mbt213634-bib-0044] Heung, L.J. , Luberto, C. , and Del Poeta, M. (2006) Role of sphingolipids in microbial pathogenesis. Infect Immun 74: 28–39. 16368954 10.1128/IAI.74.1.28-39.2006 PMC1346627

[mbt213634-bib-0045] van den Heuvel, R.H. , Curti, B. , Vanoni, M.A. , and Mattevi, A. (2004) Glutamate synthase: a fascinating pathway from L‐glutamine to L‐glutamate. Cell Mol Life Sci 61: 669–681. 15052410 10.1007/s00018-003-3316-0 PMC11138638

[mbt213634-bib-0046] Hol, W.H. , de Boer, W. , de Hollander, M. , Kuramae, E.E. , Meisner, A. , and van der Putten, W.H. (2015) Context dependency and saturating effects of loss of rare soil microbes on plant productivity. Front Plant Sci 6: 485. 26175749 10.3389/fpls.2015.00485 PMC4485053

[mbt213634-bib-0047] Huang, S. , Chen, X. , Zhong, X. , Li, M. , Ao, K. , Huang, J. , and Li, X. (2016) Plant TRAF proteins regulate NLR immune receptor turnover. Cell Host Microbe 19: 204–215. 26867179 10.1016/j.chom.2016.01.005

[mbt213634-bib-0048] Hwang, S.F. , Strelkov, S.E. , Feng, J. , Gossen, B.D. , and Howard, R.J. (2012) *Plasmodiophora brassicae*: a review of an emerging pathogen of the Canadian canola (*Brassica napus*) crop. Mol Plant Pathol 13: 105–113. 21726396 10.1111/j.1364-3703.2011.00729.x PMC6638701

[mbt213634-bib-0049] Kageyama, K. , and Asano, T. (2009) Life cycle of *Plasmodiophora brassicae* . J Plant Growth Regul 28: 203–211.

[mbt213634-bib-0050] Kielbowicz‐Matuk, A. (2012) Involvement of plant C(2)H(2)‐type zinc finger transcription factors in stress responses. Plant Sci 185–186: 78–85. 10.1016/j.plantsci.2011.11.015 22325868

[mbt213634-bib-0051] King, R. , Urban, M. , Lauder, R.P. , Hawkins, N. , Evans, M. , Plummer, A. , *et al* (2017) A conserved fungal glycosyltransferase facilitates pathogenesis of plants by enabling hyphal growth on solid surfaces. PLoS Pathog 13: e1006672. 29020037 10.1371/journal.ppat.1006672 PMC5653360

[mbt213634-bib-0052] Kombrink, A. , and Thomma, B.P. (2013) LysM effectors: secreted proteins supporting fungal life. PLoS Pathog 9: e1003769. 24348247 10.1371/journal.ppat.1003769 PMC3861536

[mbt213634-bib-0053] Kong, L.A. , Yang, J. , Li, G.T. , Qi, L.L. , Zhang, Y.J. , Wang, C.F. , *et al* (2012) Different chitin synthase genes are required for various developmental and plant infection processes in the rice blast fungus *Magnaporthe oryzae* . PLoS Pathog 8: e1002526. 22346755 10.1371/journal.ppat.1002526 PMC3276572

[mbt213634-bib-0054] Lachaise, T. , Ourry, M. , Lebreton, L. , Guillerm‐Erckelboudt, A.Y. , Linglin, J. , Paty, C. , *et al* (2017) Can soil microbial diversity influence plant metabolites and life history traits of a rhizophagous insect? A demonstration in oilseed rape. Insect Sci 24: 1045–1056. 28544806 10.1111/1744-7917.12478

[mbt213634-bib-0055] Lahlali, R. , McGregor, L. , Song, T. , Gossen, B.D. , Narisawa, K. , and Peng, G. (2014) *Heteroconium chaetospira* induces resistance to clubroot via upregulation of host genes involved in jasmonic acid, ethylene, and auxin biosynthesis. PLoS One 9: e94144. 24714177 10.1371/journal.pone.0094144 PMC3979836

[mbt213634-bib-0056] Lebeis, S.L. , Paredes, S.H. , Lundberg, D.S. , Breakfield, N. , Gehring, J. , McDonald, M. , *et al* (2015) Salicylic acid modulates colonization of the root microbiome by specific bacterial taxa. Science 349: 860–864. 26184915 10.1126/science.aaa8764

[mbt213634-bib-0057] Lebreton, L. , Guillerm‐Erckelboudt, A.Y. , Gazengel, K. , Linglin, J. , Ourry, M. , Glory, P. , *et al* (2019) Temporal dynamics of bacterial and fungal communities during the infection of *Brassica rapa* roots by the protist *Plasmodiophora brassicae* . PLoS One 14: e0204195. 30802246 10.1371/journal.pone.0204195 PMC6388920

[mbt213634-bib-0058] Lee, S.O. , Choi, G.J. , Choi, Y.H. , Jang, K.S. , Park, D.J. , Kim, C.J. , and Kim, J.C. (2008) Isolation and characterization of endophytic actinomycetes from Chinese cabbage roots as antagonists to *Plasmodiophora brassicae* . J Microbiol Biotechnol 18: 1741–1746. 19047815 10.4014/jmb.0800.108

[mbt213634-bib-0059] Lemarie, S. , Robert‐Seilaniantz, A. , Lariagon, C. , Lemoine, J. , Marnet, N. , Jubault, M. , *et al* (2015) Both the jasmonic acid and the salicylic acid pathways contribute to resistance to the biotrophic clubroot agent *Plasmodiophora brassicae* in Arabidopsis. Plant Cell Physiol 56: 2158–2168. 26363358 10.1093/pcp/pcv127

[mbt213634-bib-0060] Lenth, R.V. (2016) emmeans: Estimated Marginal Means, aka Least‐Squares Means. R package version 1.4.3. URL https://CRAN.R‐project.org/package=emmeans.

[mbt213634-bib-0061] Li, L. , Long, Y. , Li, H. , and Wu, X. (2020) Comparative transcriptome analysis reveals key pathways and hub genes in rapeseed during the early stage of Plasmodiophora brassicae infection. Front Genet 10: 1275. 32010176 10.3389/fgene.2019.01275 PMC6978740

[mbt213634-bib-0062] Liu, P. , and Stajich, J.E. (2015) Characterization of the Carbohydrate Binding Module 18 gene family in the amphibian pathogen *Batrachochytrium dendrobatidis* . Fungal Genet Biol 77: 31–39. 25819009 10.1016/j.fgb.2015.03.003

[mbt213634-bib-0063] Liu, Z. , Zhang, X. , Liu, X. , Fu, C. , Han, X. , Yin, Y. , and Ma, Z. (2016) The chitin synthase FgChs2 and other FgChss co‐regulate vegetative development and virulence in *F. graminearum* . Sci Rep 6: 34975. 27725723 10.1038/srep34975 PMC5057161

[mbt213634-bib-0064] Ludwig‐Müller, J. (2008) Glucosinolates and the clubroot disease: defense compounds or auxin precursors? Phytochem Rev 8: 135–148.

[mbt213634-bib-0065] Ludwig‐Muller, J. , Julke, S. , Geiss, K. , Richter, F. , Mithofer, A. , Sola, I. , *et al* (2015) A novel methyltransferase from the intracellular pathogen *Plasmodiophora brassicae* methylates salicylic acid. Mol Plant Pathol 16: 349–364. 25135243 10.1111/mpp.12185 PMC6638400

[mbt213634-bib-0066] Lugtenberg, B.J. , Caradus, J.R. , and Johnson, L.J. (2016) Fungal endophytes for sustainable crop production. FEMS Microbiol Ecol 92. 10.1093/femsec/fiw194 27624083

[mbt213634-bib-0067] Lundberg, D.S. , Lebeis, S.L. , Paredes, S.H. , Yourstone, S. , Gehring, J. , Malfatti, S. , *et al* (2012) Defining the core *Arabidopsis thaliana* root microbiome. Nature 488: 86–90. 22859206 10.1038/nature11237 PMC4074413

[mbt213634-bib-0068] Luo, Y. , Dong, D. , Gou, Z. , Wang, X. , Jiang, H. , Yan, Y. , *et al* (2017) Isolation and characterization of *Zhihengliuella aestuarii* B18 suppressing clubroot on *Brassica juncea* var. tumida Tsen. Eur J Plant Pathol 150: 213–222.

[mbt213634-bib-0069] Mahoney, A.K. , Yin, C. , and Hulbert, S.H. (2017) Community structure, species variation, and potential functions of rhizosphere‐associated bacteria of different winter wheat (*Triticum aestivum*) Cultivars. Front Plant Sci 8: 132. 28243246 10.3389/fpls.2017.00132 PMC5303725

[mbt213634-bib-0070] Malinowski, R. , Novák, O. , Borhan, M.H. , Spíchal, L. , Strnad, M. , and Rolfe, S.A. (2016) The role of cytokinins in clubroot disease. Eur J Plant Pathol 145: 543–557.

[mbt213634-bib-0071] Mallon, C.A. , Le Roux, X. , van Doorn, G.S. , Dini‐Andreote, F. , Poly, F. , and Salles, J.F. (2018) The impact of failure: unsuccessful bacterial invasions steer the soil microbial community away from the invader's niche. ISME J 12: 728–741. 29374268 10.1038/s41396-017-0003-y PMC5864238

[mbt213634-bib-0072] Manzanares‐Dauleux, M.J. , Divaret, I. , Baron, F. , and Thomas, G. (2000) Evaluation of French *Brassica oleracea* landraces for resistance to *Plasmodiophora brassicae* . Euphytica 113: 211–218.

[mbt213634-bib-0073] Marowa, P. , Ding, A. , and Kong, Y. (2016) Expansins: roles in plant growth and potential applications in crop improvement. Plant Cell Rep 35: 949–965. 26888755 10.1007/s00299-016-1948-4 PMC4833835

[mbt213634-bib-0074] Mendes, R. , Kruijt, M. , de Bruijn, I. , Dekkers, E. , van der Voort, M. , Schneider, J.H.M. , *et al* (2011) Deciphering the Rhizosphere microbiome for disease‐suppressive bacteria. Science 332: 1097–1100. 21551032 10.1126/science.1203980

[mbt213634-bib-0075] Mendes, R. , Garbeva, P. , and Raaijmakers, J.M. (2013) The rhizosphere microbiome: significance of plant beneficial, plant pathogenic, and human pathogenic microorganisms. FEMS Microbiol Rev 37: 634–663. 23790204 10.1111/1574-6976.12028

[mbt213634-bib-0076] Monod, M. , Capoccia, S. , Lechenne, B. , Zaugg, C. , Holdom, M. , and Jousson, O. (2002) Secreted proteases from pathogenic fungi. Int J Med Microbiol 292: 405–419. 12452286 10.1078/1438-4221-00223

[mbt213634-bib-0077] Muller, D.B. , Vogel, C. , Bai, Y. , and Vorholt, J.A. (2016) The plant microbiota: systems‐level insights and perspectives. Annu Rev Genet 50: 211–234. 27648643 10.1146/annurev-genet-120215-034952

[mbt213634-bib-0078] Muszewska, A. , Stepniewska‐Dziubinska, M.M. , Steczkiewicz, K. , Pawlowska, J. , Dziedzic, A. , and Ginalski, K. (2017) Fungal lifestyle reflected in serine protease repertoire. Sci Rep 7: 9147. 28831173 10.1038/s41598-017-09644-w PMC5567314

[mbt213634-bib-0079] Nitzsche, R. , Gunay‐Esiyok, O. , Tischer, M. , Zagoriy, V. , and Gupta, N. (2017) A plant/fungal‐type phosphoenolpyruvate carboxykinase located in the parasite mitochondrion ensures glucose‐independent survival of *Toxoplasma gondii* . J Biol Chem 292: 15225–15239. 28726641 10.1074/jbc.M117.802702 PMC5602384

[mbt213634-bib-0080] Oh, Y. , Donofrio, N. , Pan, H. , Coughlan, S. , Brown, D.E. , Meng, S. , *et al* (2008) Transcriptome analysis reveals new insight into appressorium formation and function in the rice blast fungus *Magnaporthe oryzae* . Genome Biol 9: R85. 18492280 10.1186/gb-2008-9-5-r85 PMC2441471

[mbt213634-bib-0081] Oksanen, J. , Blanchet, G.F. , Friendly, M. , Kindt, R. , Legendre, P. , McGlinn, D. , *et al* (2019) vegan: Community Ecology Package. R package version 2.5‐6. URL https://CRAN.R‐project.org/package=vegan.

[mbt213634-bib-0082] Ourry, M. , Lebreton, L. , Chaminade, V. , Guillerm‐Erckelboudt, A.‐Y. , Hervé, M. , Linglin, J. , *et al* (2018) Influence of belowground herbivory on the dynamics of root and rhizosphere microbial communities. Front Ecol Evol 6: 91.

[mbt213634-bib-0083] Perez‐Lopez, E. , Waldner, M. , Hossain, M. , Kusalik, A.J. , Wei, Y. , Bonham‐Smith, P.C. , and Todd, C.D. (2018) Identification of *Plasmodiophora brassicae* effectors – a challenging goal. Virulence 9: 1344–1353. 30146948 10.1080/21505594.2018.1504560 PMC6177251

[mbt213634-bib-0084] Plassart, P. , Terrat, S. , Thomson, B. , Griffiths, R. , Dequiedt, S. , Lelievre, M. , *et al* (2012) Evaluation of the ISO standard 11063 DNA extraction procedure for assessing soil microbial abundance and community structure. PLoS One 7: e44279. 22984486 10.1371/journal.pone.0044279 PMC3439486

[mbt213634-bib-0085] Ploch, S. , Rose, L.E. , Bass, D. , and Bonkowski, M. (2016) High diversity revealed in leaf‐associated protists (Rhizaria: Cercozoa) of Brassicaceae. J Eukaryot Microbiol 63: 635–641. 27005328 10.1111/jeu.12314 PMC5031217

[mbt213634-bib-0086] Podolich, O. , Ardanov, P. , Zaets, I. , Pirttilä, A.M. , and Kozyrovska, N. (2014) Reviving of the endophytic bacterial community as a putative mechanism of plant resistance. Plant Soil 388: 367–377.

[mbt213634-bib-0087] Quast, C. , Pruesse, E. , Yilmaz, P. , Gerken, J. , Schweer, T. , Yarza, P. , *et al* (2013) The SILVA ribosomal RNA gene database project: improved data processing and web‐based tools. Nucleic Acids Res 41: D590–D596. 23193283 10.1093/nar/gks1219 PMC3531112

[mbt213634-bib-0088] Raaijmakers, J.M. , Paulitz, T.C. , Steinberg, C. , Alabouvette, C. , and Moënne‐Loccoz, Y. (2008) The rhizosphere: a playground and battlefield for soilborne pathogens and beneficial microorganisms. Plant Soil 321: 341–361.

[mbt213634-bib-0089] Rolfe, S.A. , Strelkov, S.E. , Links, M.G. , Clarke, W.E. , Robinson, S.J. , Djavaheri, M. , *et al* (2016) The compact genome of the plant pathogen *Plasmodiophora brassicae* is adapted to intracellular interactions with host *Brassica spp* . BMC Genom 17: 272. 10.1186/s12864-016-2597-2 PMC4815078 27036196

[mbt213634-bib-0090] Rolli, E. , Marasco, R. , Vigani, G. , Ettoumi, B. , Mapelli, F. , Deangelis, M.L. , *et al* (2015) Improved plant resistance to drought is promoted by the root‐associated microbiome as a water stress‐dependent trait. Environ Microbiol 17: 316–331. 24571749 10.1111/1462-2920.12439

[mbt213634-bib-0091] Santhanam, R. , Luu, V.T. , Weinhold, A. , Goldberg, J. , Oh, Y. , and Baldwin, I.T. (2015) Native root‐associated bacteria rescue a plant from a sudden‐wilt disease that emerged during continuous cropping. Proc Natl Acad Sci USA 112: E5013–5020. 26305938 10.1073/pnas.1505765112 PMC4568709

[mbt213634-bib-0092] Schaeffer, A. , Bronner, R. , Benveniste, P. , and Schaller, H. (2001) The ratio of campesterol to sitosterol that modulates growth in *Arabidopsis* is controlled by *STEROL METHYLTRANSFERASE 2;1* . Plant J 25: 605–615. 11319028 10.1046/j.1365-313x.2001.00994.x

[mbt213634-bib-0093] Schuller, A. , Kehr, J. , and Ludwig‐Muller, J. (2014) Laser microdissection coupled to transcriptional profiling of Arabidopsis roots inoculated by *Plasmodiophora brassicae* indicates a role for brassinosteroids in clubroot formation. Plant Cell Physiol 55: 392–411. 24285749 10.1093/pcp/pct174

[mbt213634-bib-0094] Schwelm, A. , Dixelius, C. , and Ludwig‐Müller, J. (2015a) New kid on the block – the clubroot pathogen genome moves the plasmodiophorids into the genomic era. Eur J Plant Pathol 145: 531–542.

[mbt213634-bib-0095] Schwelm, A. , Fogelqvist, J. , Knaust, A. , Julke, S. , Lilja, T. , Bonilla‐Rosso, G. , *et al* (2015b) The *Plasmodiophora brassicae* genome reveals insights in its life cycle and ancestry of chitin synthases. Sci Rep 5: 11153. 26084520 10.1038/srep11153 PMC4471660

[mbt213634-bib-0096] Sedbrook, J.C. , Carroll, K.L. , Hung, K.F. , Masson, P.H. , and Somerville, C.R. (2002) The Arabidopsis SKU5 gene encodes an extracellular glycosyl phosphatidylinositol‐anchored glycoprotein involved in directional root growth. Plant Cell 14: 1635–1648. 12119380 10.1105/tpc.002360 PMC150712

[mbt213634-bib-0097] Shakeel, Q. , Lyu, A. , Zhang, J. , Wu, M. , Chen, S. , Chen, W. , *et al* (2016) Optimization of the cultural medium and conditions for production of antifungal substances by *Streptomyces platensis* 3–10 and evaluation of its efficacy in suppression of clubroot disease ( *Plasmodiophora brassicae* ) of oilseed rape. Biol Control 101: 59–68.

[mbt213634-bib-0098] Shnaiderman, C. , Miyara, I. , Kobiler, I. , Sherman, A. , and Prusky, D. (2013) Differential activation of ammonium transporters during the accumulation of ammonia by *Colletotrichum gloeosporioides* and its effect on appressoria formation and pathogenicity. Mol Plant Microbe Interact 26: 345–355. 23387470 10.1094/MPMI-07-12-0170-R

[mbt213634-bib-0099] Showalter, A.M. (2001) (2001) Arabinogalactan‐proteins: structure, expression and function. Cell Mol Life Sci 58(10): 1399–1417. 11693522 10.1007/PL00000784 PMC11337269

[mbt213634-bib-0100] Siemens, J. , Keller, I. , Sarx, J. , Kunz, S. , Schuller, A. , Nagel, W. , *et al* (2006) Transcriptome analysis of *Arabidopsis* Clubroots indicate a key role for cytokinins in disease development. Mol Plant‐Microbe Interact 19: 480–494. 16673935 10.1094/MPMI-19-0480

[mbt213634-bib-0101] Singh, K. , Winter, M. , Zouhar, M. , and Rysanek, P. (2018) Cyclophilins: less studied proteins with critical roles in pathogenesis. Phytopathology 108: 6–14. 28643580 10.1094/PHYTO-05-17-0167-RVW

[mbt213634-bib-0102] Some, A. , Manzanares, M.J. , Laurens, F. , Baron, F. , Thomas, G. , and Rouxel, F. (1996) Variation for virulence on *Brassica napus* L amongst *Plasmodiophora brassicae* collections from France and derived single‐spore isolates. Plant Pathol 45: 432–439.

[mbt213634-bib-0103] Song, J.B. , Gao, S. , Sun, D. , Li, H. , Shu, X.X. , and Yang, Z.M. (2013) miR394 and LCR are involved in Arabidopsis salt and drought stress responses in an abscisic acid‐dependent manner. BMC Plant Biol 13: 210. 24330668 10.1186/1471-2229-13-210 PMC3870963

[mbt213634-bib-0104] Terrat, S. , Christen, R. , Dequiedt, S. , Lelievre, M. , Nowak, V. , Regnier, T. , *et al* (2012) Molecular biomass and MetaTaxogenomic assessment of soil microbial communities as influenced by soil DNA extraction procedure. Microb Biotechnol 5: 135–141. 21989224 10.1111/j.1751-7915.2011.00307.x PMC3815280

[mbt213634-bib-0105] Terrat, S. , Dequiedt, S. , Horrigue, W. , Lelievre, M. , Cruaud, C. , Saby, N.P. , *et al* (2015) Improving soil bacterial taxa‐area relationships assessment using DNA meta‐barcoding. Heredity 114: 468–475. 25293875 10.1038/hdy.2014.91 PMC4815512

[mbt213634-bib-0106] Tommerup, I.C. , and Ingram, D.S. (1971) Life‐cycle of *Plasmodiophora brassicae* woron. in *brassica* tissue cultures and in intact roots. New Phytol 70: 327–332.

[mbt213634-bib-0107] Turner, T.R. , James, E.K. , and Poole, P.S. (2013) The plant microbiome. Genome Biol 14: 10. 10.1186/gb-2013-14-6-209 PMC3706808 23805896

[mbt213634-bib-0108] Vacher, C. , Hampe, A. , Porté, A.J. , Sauer, U. , Compant, S. , and Morris, C.E. (2016) The Phyllosphere: microbial jungle at the plant‐climate interface. Annu Rev Ecol Evol Syst 47: 1–24.

[mbt213634-bib-0109] Van Lijsebettens, M. , and Grasser, K.D. (2014) Transcript elongation factors: shaping transcriptomes after transcript initiation. Trends Plant Sci 19: 717–726. 25131948 10.1016/j.tplants.2014.07.002

[mbt213634-bib-0110] Vandenkoornhuyse, P. , Quaiser, A. , Duhamel, M. , Le Van, A. , and Dufresne, A. (2015) The importance of the microbiome of the plant holobiont. New Phytol 206: 1196–1206. 25655016 10.1111/nph.13312

[mbt213634-bib-0111] Vandepoele, K. , Raes, J. , De Veylder, L. , Rouze, P. , Rombauts, S. , and Inze, D. (2002) Genome‐wide analysis of core cell cycle genes in Arabidopsis. Plant Cell 14: 903–916. 11971144 10.1105/tpc.010445 PMC150691

[mbt213634-bib-0112] Vannier, N. , Agler, M. , and Hacquard, S. (2019) Microbiota‐mediated disease resistance in plants. PLoS Pathog 15: e1007740. 31194849 10.1371/journal.ppat.1007740 PMC6564022

[mbt213634-bib-0113] Varanini, Z. , Cesco, S. , Tomasi, N. , Pinton, R. , Guzzo, F. , Zamboni, A. , *et al* (2018) Nitrate induction and physiological responses of two maize lines differing in nitrogen use efficiency: effects on N availability, microbial diversity and enzyme activity in the rhizosphere. Plant Soil 422: 331–347.

[mbt213634-bib-0114] Vayssier‐Taussat, M. , Albina, E. , Citti, C. , Cosson, J.F. , Jacques, M.A. , Lebrun, M.H. , *et al* (2014) Shifting the paradigm from pathogens to pathobiome: new concepts in the light of meta‐omics. Front Cell Infect Microbiol 4: 29. 24634890 10.3389/fcimb.2014.00029 PMC3942874

[mbt213634-bib-0115] Vylkova, S. (2017) Environmental pH modulation by pathogenic fungi as a strategy to conquer the host. PLoS Pathog 13: e1006149. 28231317 10.1371/journal.ppat.1006149 PMC5322887

[mbt213634-bib-0116] Walters, W.A. , Jin, Z. , Youngblut, N. , Wallace, J.G. , Sutter, J. , Zhang, W. , *et al* (2018) Large‐scale replicated field study of maize rhizosphere identifies heritable microbes. Proc Natl Acad Sci USA 115: 7368–7373. 29941552 10.1073/pnas.1800918115 PMC6048482

[mbt213634-bib-0117] Westermann, A.J. , Gorski, S.A. , and Vogel, J. (2012) Dual RNA‐seq of pathogen and host. Nat Rev Microbiol 10: 618–630. 22890146 10.1038/nrmicro2852

[mbt213634-bib-0118] Wolf, T. , Kammer, P. , Brunke, S. , and Linde, J. (2018) Two's company: studying interspecies relationships with dual RNA‐seq. Curr Opin Microbiol 42: 7–12. 28957710 10.1016/j.mib.2017.09.001

[mbt213634-bib-0119] Xia, Y. , Suzuki, H. , Borevitz, J. , Blount, J. , Guo, Z. , Patel, K. , *et al* (2004) An extracellular aspartic protease functions in *Arabidopsis* disease resistance signaling. EMBO J 23: 980–988. 14765119 10.1038/sj.emboj.7600086 PMC380998

[mbt213634-bib-0120] Xu, S.J. , Hong, S.J. , Choi, W. , and Kim, B.S. (2014) Antifungal activity of *Paenibacillus kribbensis* Strain T‐9 isolated from soils against several plant pathogenic fungi. Plant Pathol J 30: 102–108. 25288992 10.5423/PPJ.OA.05.2013.0052 PMC4174836

[mbt213634-bib-0121] Yan, Y. , Kuramae, E.E. , de Hollander, M. , Klinkhamer, P.G. , and van Veen, J.A. (2017) Functional traits dominate the diversity‐related selection of bacterial communities in the rhizosphere. ISME J 11: 56–66. 27482928 10.1038/ismej.2016.108 PMC5315473

[mbt213634-bib-0122] Yao, H. , and Wu, F. (2010) Soil microbial community structure in cucumber rhizosphere of different resistance cultivars to fusarium wilt. FEMS Microbiol Ecol 72: 456–463. 20370829 10.1111/j.1574-6941.2010.00859.x

[mbt213634-bib-0123] Yin, Y. , Vafeados, D. , Tao, Y. , Yoshida, S. , Asami, T. , and Chory, J. (2005) A new class of transcription factors mediates brassinosteroid‐regulated gene expression in *Arabidopsis* . Cell 120: 249–259. 15680330 10.1016/j.cell.2004.11.044

[mbt213634-bib-0124] Yu, F. , Wang, S. , Zhang, W. , Tang, J. , Wang, H. , Yu, L. , *et al* (2019) Genome‐wide identification of genes encoding putative secreted E3 ubiquitin ligases and functional characterization of PbRING1 in the biotrophic protist *Plasmodiophora brassicae* . Curr Genet 65: 1355–1365. 31087129 10.1007/s00294-019-00989-5

[mbt213634-bib-0125] Yuan, J. , Zhao, J. , Wen, T. , Zhao, M. , Li, R. , Goossens, P. , *et al* (2018) Root exudates drive the soil‐borne legacy of aboveground pathogen infection. Microbiome 6: 156. 30208962 10.1186/s40168-018-0537-x PMC6136170

[mbt213634-bib-0126] Zeng, L.R. , Park, C.H. , Venu, R.C. , Gough, J. , and Wang, G.L. (2008) Classification, expression pattern, and E3 ligase activity assay of rice U‐box‐containing proteins. Mol Plant 1: 800–815. 19825583 10.1093/mp/ssn044

[mbt213634-bib-0127] Zhang, D. , Burroughs, A.M. , Vidal, N.D. , Iyer, L.M. , and Aravind, L. (2016) Transposons to toxins: the provenance, architecture and diversification of a widespread class of eukaryotic effectors. Nucleic Acids Res 44: 3513–3533. 27060143 10.1093/nar/gkw221 PMC4857004

[mbt213634-bib-0128] Zhao, Y. , Hu, Y. , Dai, M. , Huang, L. , and Zhou, D.X. (2009) The WUSCHEL‐related homeobox gene *WOX11* is required to activate shoot‐borne crown root development in rice. Plant Cell 21: 736–748. 19258439 10.1105/tpc.108.061655 PMC2671696

[mbt213634-bib-0129] Zhao, J. , Wu, Y.‐X. , Ho, H.‐H. , Chen, Z.‐J. , Li, X.‐Y. , and He, Y.‐Q. (2016) PBT1, a novel antimicrobial protein from the biocontrol agent *Bacillus subtilis* XF‐1 against *Plasmodiophora brassicae* . Eur J Plant Pathol 145: 583–590.

[mbt213634-bib-0130] Zhao, Y. , Gao, Z. , Tian, B. , Bi, K. , Chen, T. , Liu, H. , *et al* (2017) Endosphere microbiome comparison between symptomatic and asymptomatic roots of *Brassica napus* infected with *Plasmodiophora brassicae* . PLoS One 12: e0185907. 29065162 10.1371/journal.pone.0185907 PMC5655474

[mbt213634-bib-0131] Zhou, L. , Li, M. , Yang, J. , Wei, L. , and Ji, G. (2014) Draft genome sequence of antagonistic agent *Lysobacter antibioticus* 13–6. Genome Announc 2: e00566‐00514. 25301638 10.1128/genomeA.00566-14 PMC4192370

[mbt213634-bib-0132] Zhu, S. , Vivanco, J.M. , and Manter, D.K. (2016) Nitrogen fertilizer rate affects root exudation, the rhizosphere microbiome and nitrogen‐use‐efficiency of maize. Appl Soil Ecol 107: 324–333.

