# Supplementary material for: Soil microbiota influences clubroot disease by modulating Plasmodiophora brassicae and Brassica napus transcriptomes
Source: Microb Biotechnol. 2020 Jul 19;13(5):1648–72. doi: 10.1111/1751-7915.13634 (PMC7415369; doi:10.1111/1751-7915.13634)
Supplement: Supplementary file 1 — Fig. S1. Microbiological follow up based on the Colony Forming Units (CFU) method during the incubation period for bacteria (A) and fungi (B). H, High diversity modality; M, Medium diversity modality; L, Low diversity modality. [file MBT2-13-1648-s001.pdf]

**A**

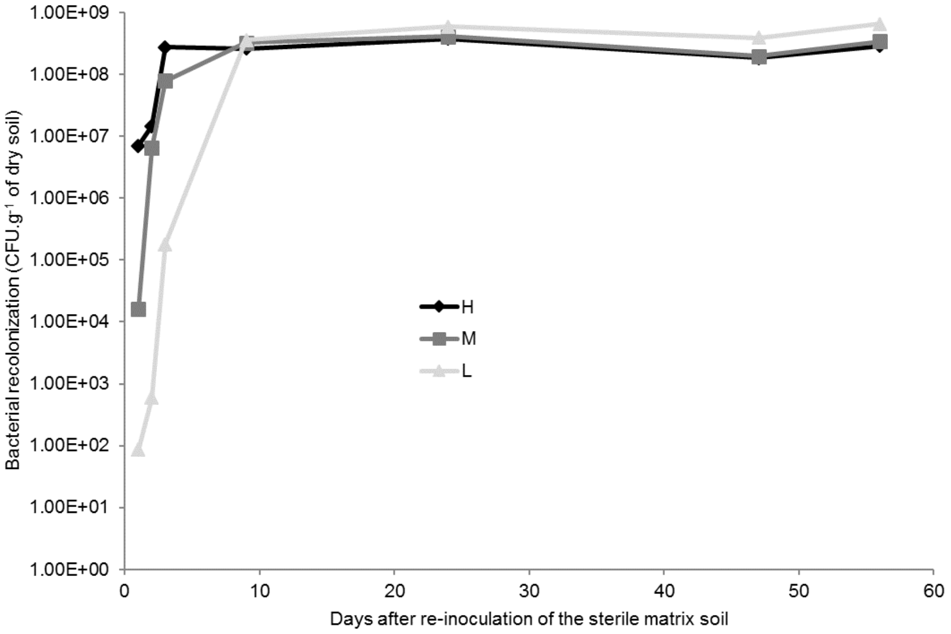


**B**

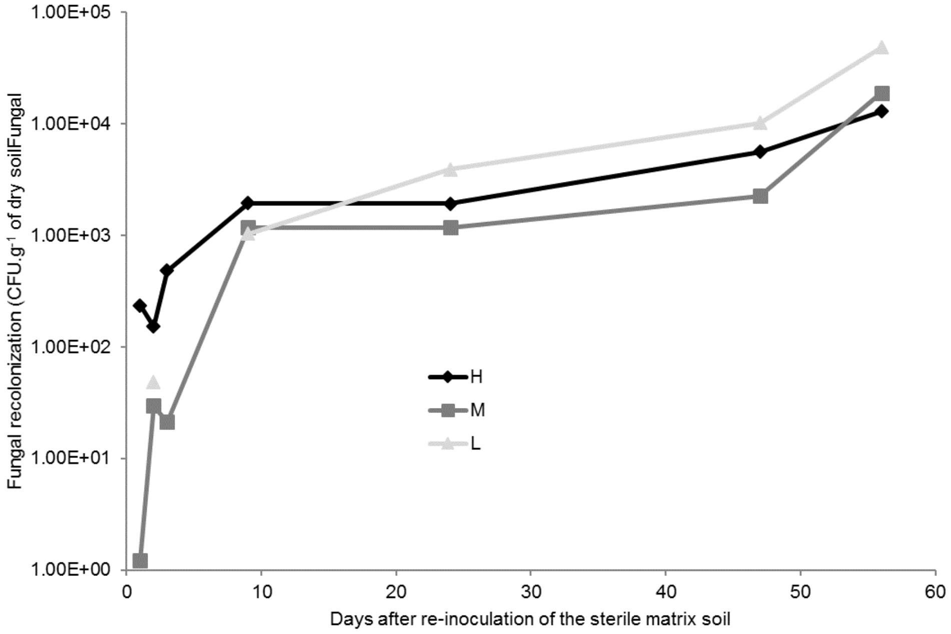


S1 Fig. Microbiological follow up based on the Colony Forming Units (CFU) method during the incubation period for bacteria (A) and fungi (B). H, High diversity modality; M, Medium diversity modality; L, Low diversity modality.
