# Supplementary material for: Soil microbiota influences clubroot disease by modulating Plasmodiophora brassicae and Brassica napus transcriptomes
Source: Microb Biotechnol. 2020 Jul 19;13(5):1648–72. doi: 10.1111/1751-7915.13634 (PMC7415369; doi:10.1111/1751-7915.13634)
Supplement: Supplementary file 2 — Fig. S2. Description of the main bacterial and fungal composition in the three soils. Average relative abundance (RA ± SEM) of the most abundant bacterial phyla (A), genera (B), OTUs (C), and fungal phyla (D), genera (E), OTUs (F) are shown in High (H), Medium (M) and Low (L) soil microbial diversities. For each soil, the number of replicates is n = 3. [file MBT2-13-1648-s002.pdf]

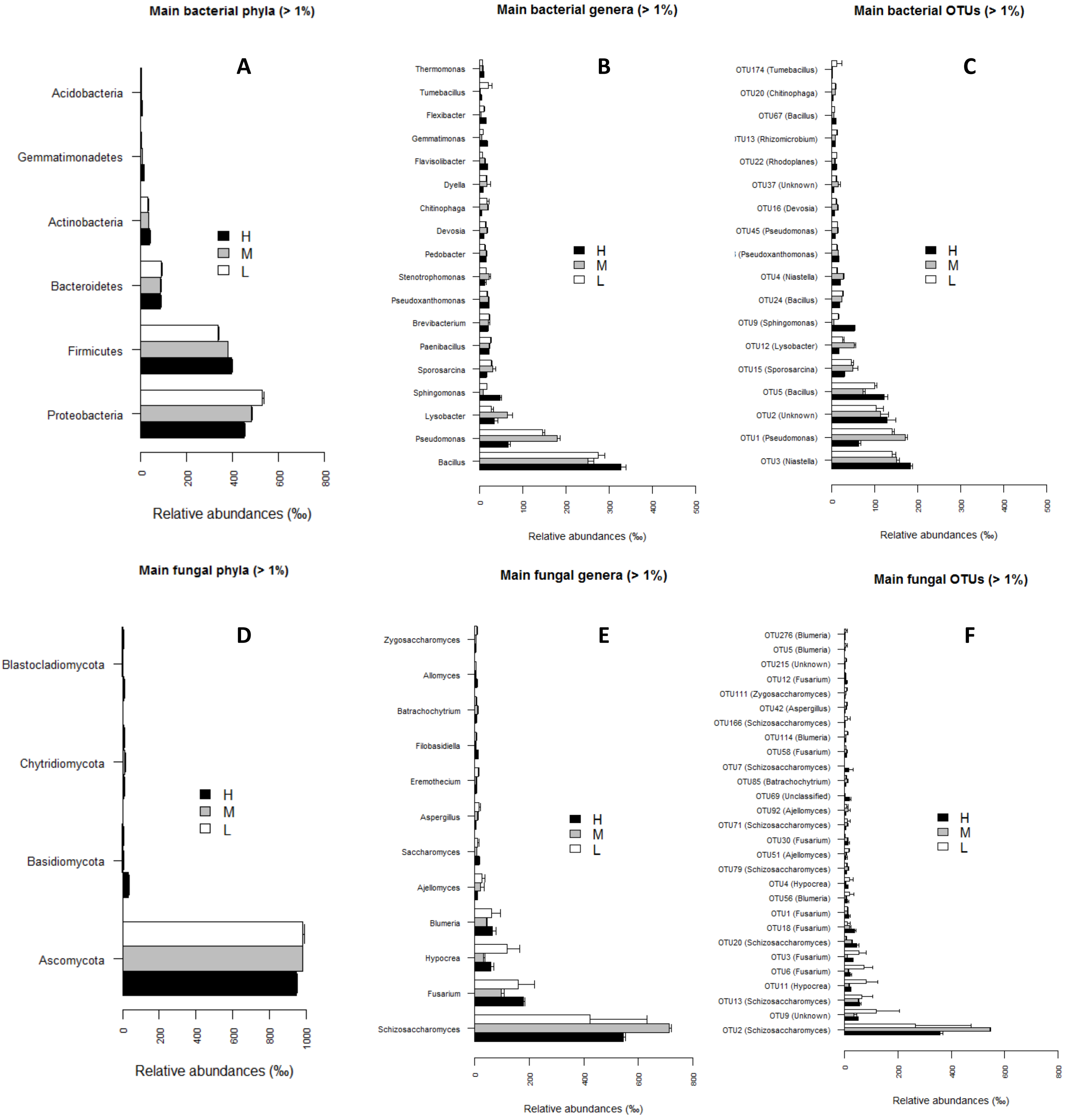

S2 Fig. Description of the main bacterial and fungal composition in the three soils. Average relative abundance (RA ± SEM) of the most abundant bacterial phyla (A), genera (B), OTUs (C), and fungal phyla (D), genera (E), OTUs (F) are shown in High (H), Medium (M) and Low (L) soil microbial diversities. For each soil, the number of replicates is n=3.
