# Supplementary material for: Soil microbiota influences clubroot disease by modulating Plasmodiophora brassicae and Brassica napus transcriptomes
Source: Microb Biotechnol. 2020 Jul 19;13(5):1648–72. doi: 10.1111/1751-7915.13634 (PMC7415369; doi:10.1111/1751-7915.13634)
Supplement: Supplementary file 3 — Fig. S3. Overview of all P. brassicae transcriptome samples. A. Heatmaps of P. brassicae gene expression based on normalized data of expression values. The heatmaps are based on total reads counts for P. brassicae at Ti and Tf for the 3 microbial soil diversities (H, High; M, Medium, L, Low), the two plant genotypes (T, Tenor; Y, Yudal) and correspond to the mean of the three replicates. B. Hierarchical Cluster Analysis (HCA) of the filtered and normalized counts in the dual‐RNAseq analysis. The analyses are shown for P. brassicae reads at Ti and Tf for the 3 soil microbial diversities (H, High; M, Medium; L, Low), the two plant genotypes (T, Tenor; Y, Yudal), and the three replicates (a, b, c). [file MBT2-13-1648-s003.pdf]

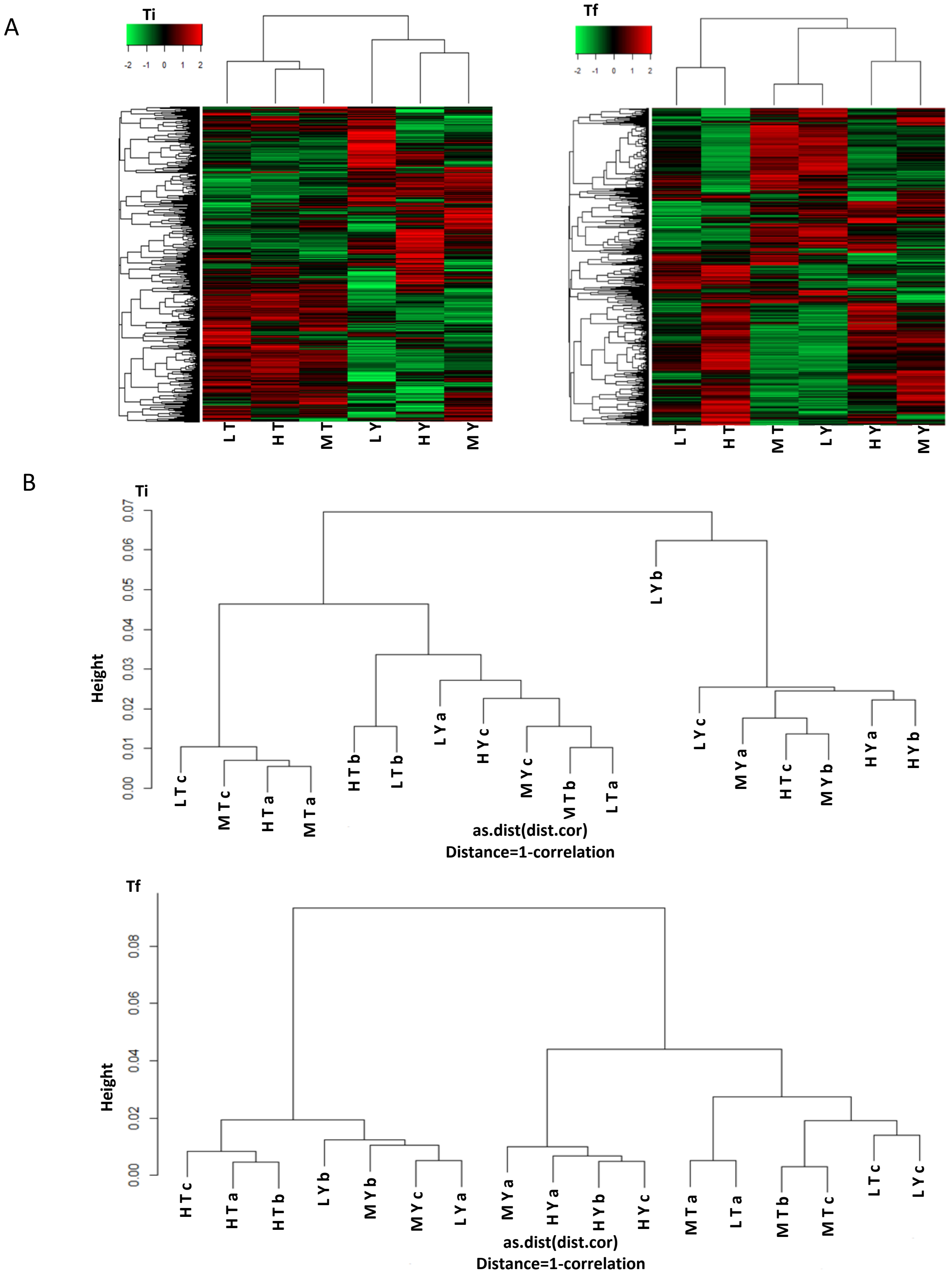

S3 Fig. Overview of all *P. brassicae* transcriptome samples. A. Heatmaps of *P. brassicae* gene expression based on normalized data of expression values. The heatmaps are based on total reads counts for *P. brassicae* at Ti and Tf for the 3 microbial soil diversities (H, High; M, Medium, L, Low), the two plant genotypes (T, Tenor; Y, Yudal) and correspond to the mean of the three replicates. B. Hierarchical Cluster Analysis (HCA) of the filtered and normalized counts in the dual-RNAseq analysis. The analyses are shown for *P. brassicae* reads at Ti and Tf for the 3 soil microbial diversities (H, High; M, Medium; L, Low), the two plant genotypes (T, Tenor; Y, Yudal), and the three replicates (a, b, c).
