# Supplementary material for: Soil microbiota influences clubroot disease by modulating Plasmodiophora brassicae and Brassica napus transcriptomes
Source: Microb Biotechnol. 2020 Jul 19;13(5):1648–72. doi: 10.1111/1751-7915.13634 (PMC7415369; doi:10.1111/1751-7915.13634)
Supplement: Supplementary file 4 — Fig. S4. Overview of all B. napus transcriptome samples. Hierarchical Cluster Analysis (HCA) of the filtered and normalized counts in the dual‐RNAseq analysis in healthy plants (A) and infected plants (B). The analyses are shown for B. napus reads at Ti and Tf, for the 3 soil microbial diversities (H, High; M, Medium; L, Low), the two plant genotypes (T, Tenor; Y, Yudal), and the three replicates (a, b, c). [file MBT2-13-1648-s004.pdf]

A. Healthy plants

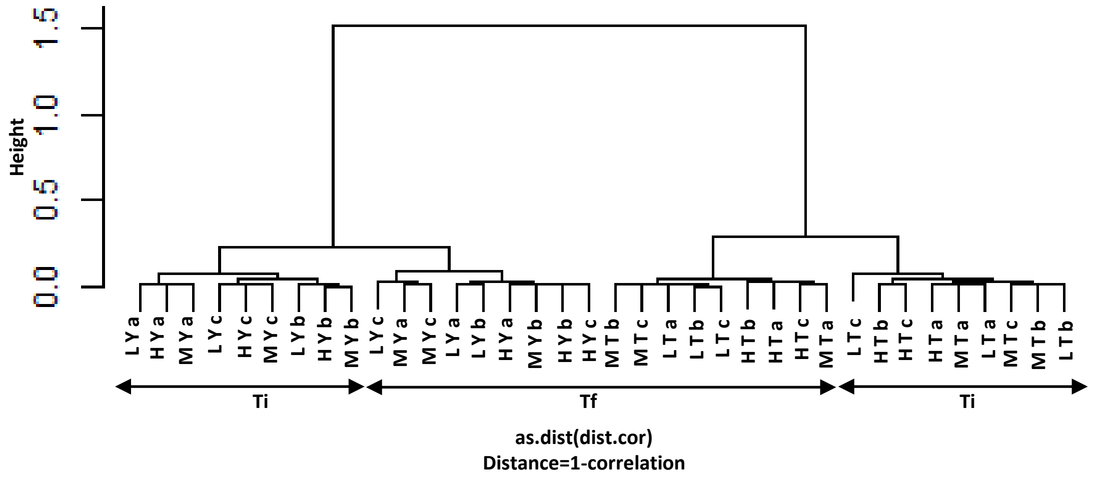

B. Infected plants

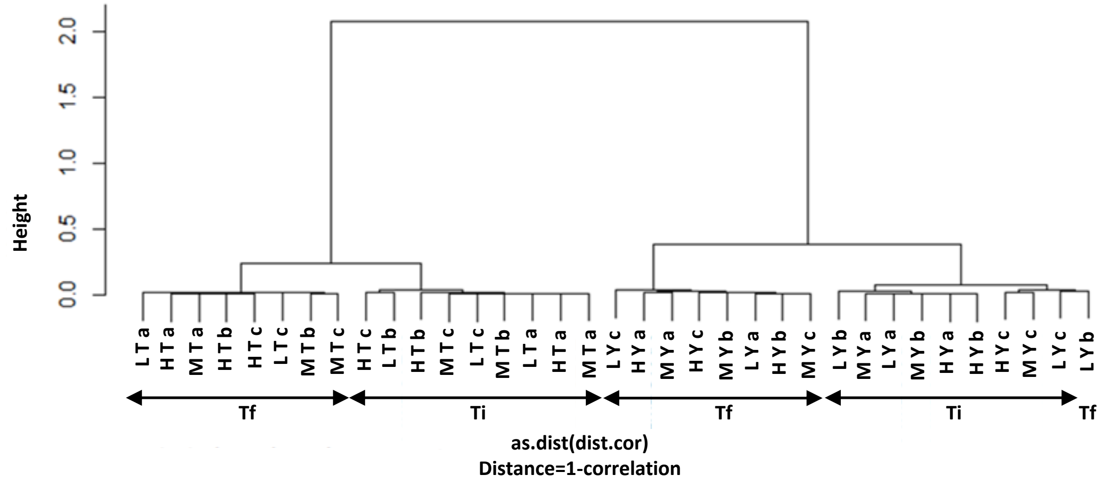

S4 Fig. Overview of all *B. napus* transcriptome samples. Hierarchical Cluster Analysis (HCA) of the filtered and normalized counts in the dual-RNAseq analysis in healthy plants (A) and infected plants (B). The analyses are shown for *B. napus* reads at Ti and Tf, for the 3 soil microbial diversities (H, High; M, Medium; L, Low), the two plant genotypes (T, Tenor; Y, Yudal), and the three replicates (a, b, c).
