# Supplementary material for: Soil microbiota influences clubroot disease by modulating Plasmodiophora brassicae and Brassica napus transcriptomes
Source: Microb Biotechnol. 2020 Jul 19;13(5):1648–72. doi: 10.1111/1751-7915.13634 (PMC7415369; doi:10.1111/1751-7915.13634)
Supplement: Supplementary file 5 — Fig. S5. Number of B. napus differentially expressed genes (DEGs) in function of the host plant genotype for each soil microbial diversity level when not infected (A) or infected by P. brassicae (B). The Venn diagram shows the number of significantly DEGs (P < 0.05) according to the host B. napus genotypes (T, Tenor; Y, Yudal) infected or not, for each soil microbial diversity level (H, High; M, Medium; L, Low) at the sampling dates Ti and Tf. [file MBT2-13-1648-s005.pdf]

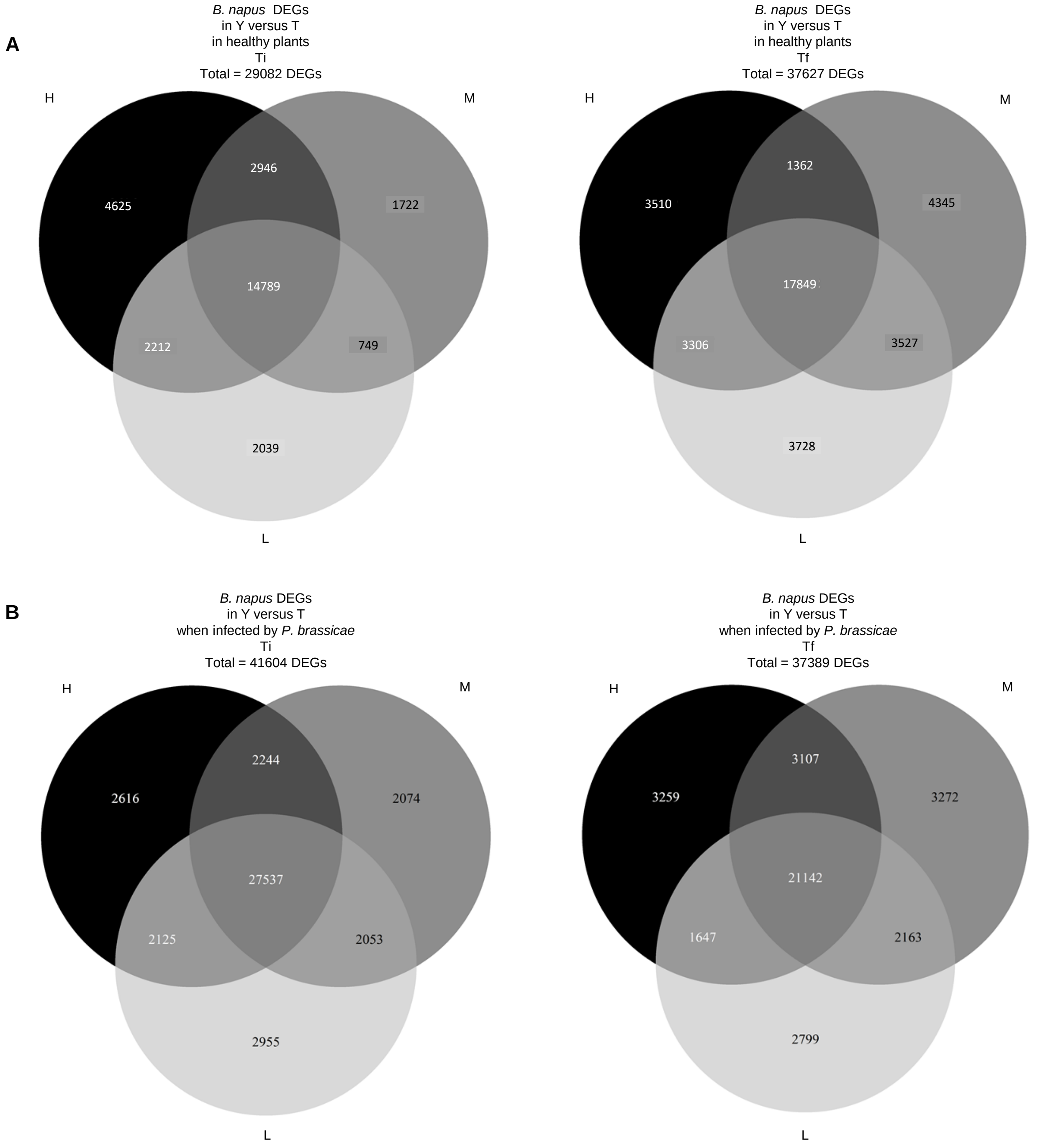

S5 Fig. Number of *B. napus d*ifferentially expressed genes (DEGs) in function of the host plant genotype for each soil microbial diversity level when not infected (A) or infected by *P. brassicae* (B). The Venn diagram shows the number of significantly DEGs ($P < 0.05$) according to the host *B. napus* genotypes (T, Tenor; Y, Yudal) infected or not, for each soil microbial diversity level (H, High; M, Medium; L, Low) at the sampling dates Ti and Tf.
