# Supplementary material for: Soil microbiota influences clubroot disease by modulating Plasmodiophora brassicae and Brassica napus transcriptomes
Source: Microb Biotechnol. 2020 Jul 19;13(5):1648–72. doi: 10.1111/1751-7915.13634 (PMC7415369; doi:10.1111/1751-7915.13634)
Supplement: Supplementary file 6 — Fig. S6. Number of B. napus differentially expressed genes (DEGs) in function of the interaction stage for each soil microbial diversity level. The Venn diagrams show the total number of significantly DEGs (P < 0.05) in the B. napus genotypes (T, Tenor; Y, Yudal), healthy (A) or infected by P. brassicae (B), at each soil microbial diversity level (H, High; M, Medium; L, Low), between Ti and Tf. [file MBT2-13-1648-s006.pdf]

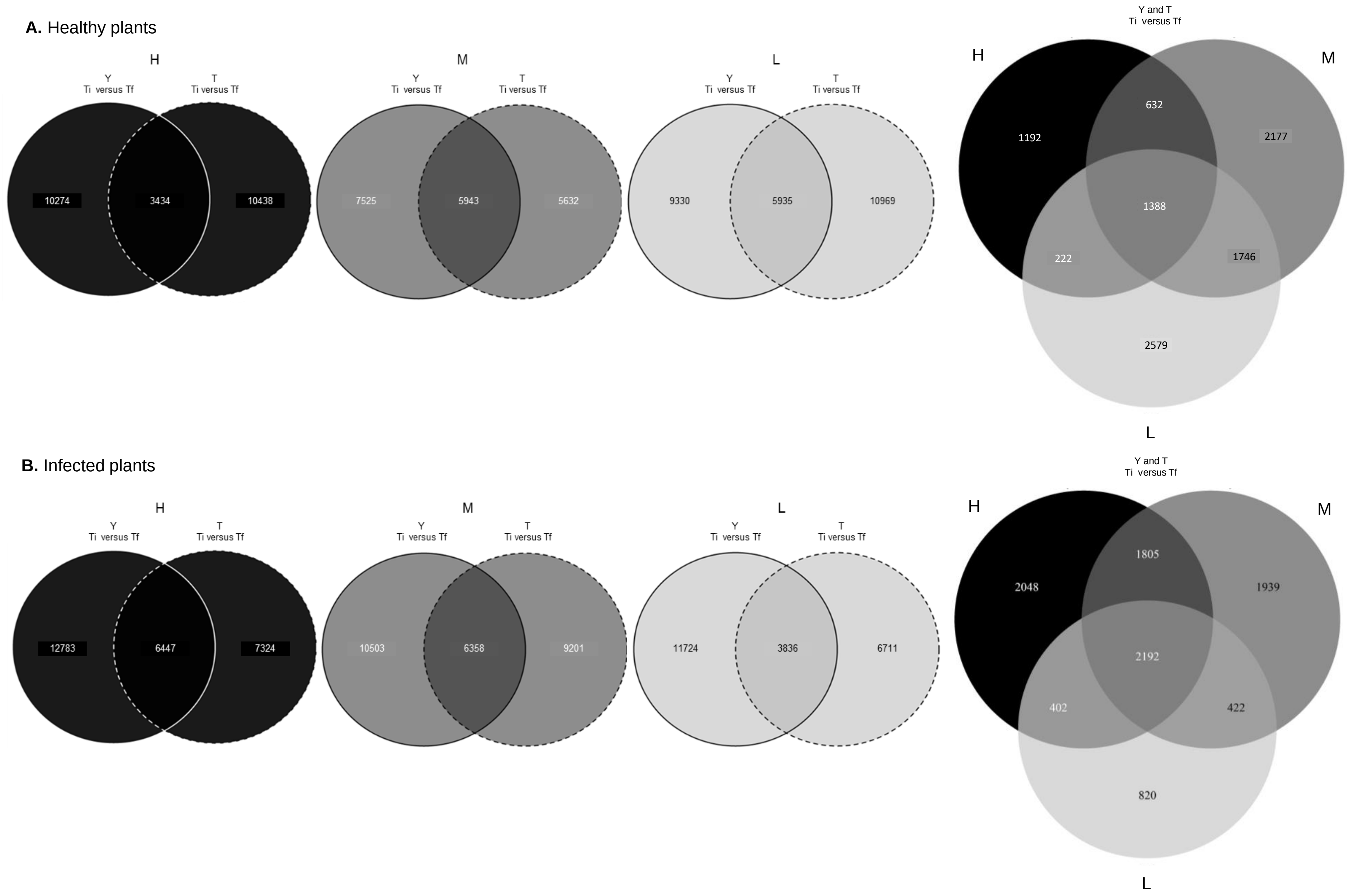

S6 Fig. Number of *B. napus* differentially expressed genes (DEGs) in function of the interaction stage for each soil microbial diversity level. The Venn diagrams show the total number of significantly DEGs ($P < 0.05$) in the *B. napus* genotypes (T, Tenor; Y, Yudal), healthy (A) or infected by *P. brassicae* (B), at each soil microbial diversity level (H, High; M, Medium; L, Low), between Ti and Tf.
