# Supplementary material for: Soil microbiota influences clubroot disease by modulating Plasmodiophora brassicae and Brassica napus transcriptomes
Source: Microb Biotechnol. 2020 Jul 19;13(5):1648–72. doi: 10.1111/1751-7915.13634 (PMC7415369; doi:10.1111/1751-7915.13634)
Supplement: Supplementary file 7 — Fig. S7. Differentially expressed genes (DEGs) in both infected B. napus genotypes according to the infection’s stage whatever the soil microbial diversity. A. The Venn diagram shows the number of significantly DEGs (P < 0.05) common in both B. napus genotypes (T, Tenor; Y, Yudal), and common in the three soil microbial diversity levels (H, High; M, Medium; L, Low), which are down (<) or up (>) regulated at Ti compared to Tf. B. Heatmaps of the 40 genes surrounded by a grey circle in the figure A. The expression is based on normalized data of expression values (T, Tenor; Y, Yudal; H, M, L, High, Medium, Low soil microbial diversity levels). [file MBT2-13-1648-s007.pdf]

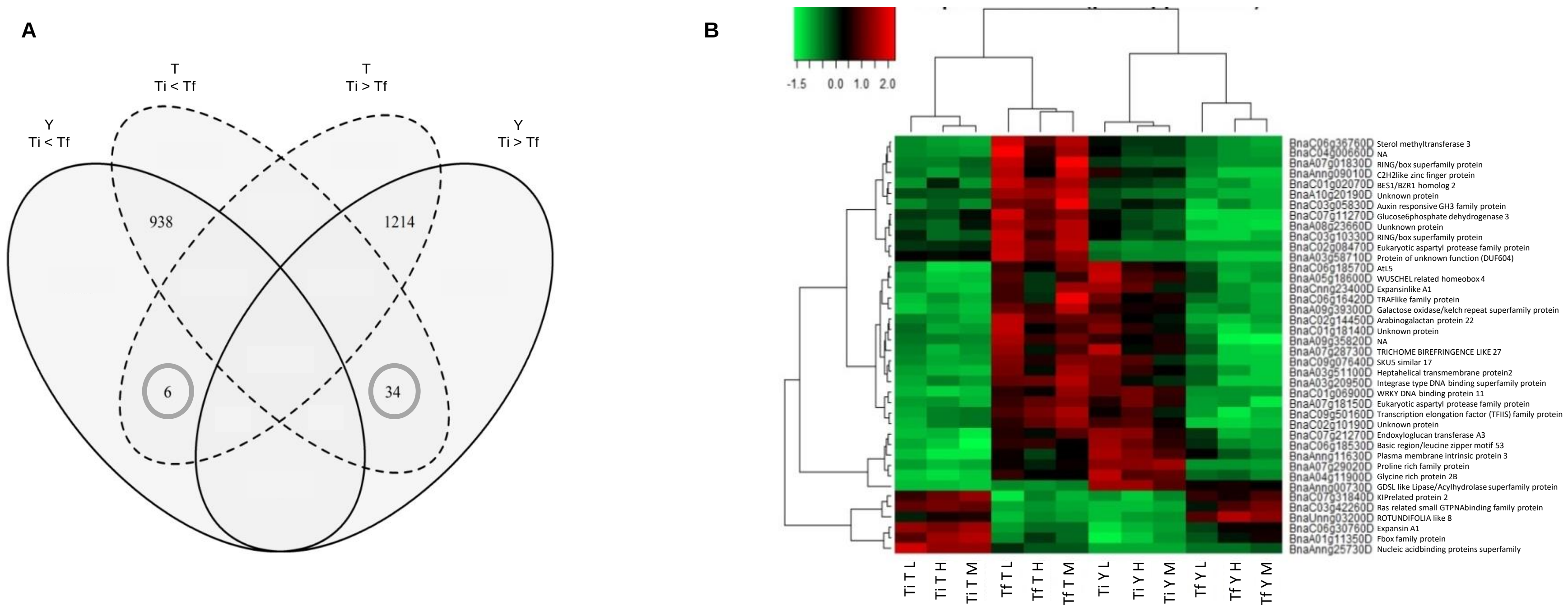

S7 Fig. Differentially expressed genes (DEGs) in both infected *B. napus* genotypes according to the infection's stage whatever the soil microbial diversity. A. The Venn diagram shows the number of significantly DEGs ($P < 0.05$) common in both *B. napus* genotypes (T, Tenor; Y, Yudal), and common in the three soil microbial diversity levels (H, High; M, Medium; L, Low), which are down (<) or up (>) regulated at Ti compared to Tf. B. Heatmaps of the 40 genes surrounded by a grey circle in the figure A. The expression is based on normalized data of expression values (T, Tenor; Y, Yudal; H, M, L, High, Medium, Low soil microbial diversity levels).
