# Supplementary material for: Soil microbiota influences clubroot disease by modulating Plasmodiophora brassicae and Brassica napus transcriptomes
Source: Microb Biotechnol. 2020 Jul 19;13(5):1648–72. doi: 10.1111/1751-7915.13634 (PMC7415369; doi:10.1111/1751-7915.13634)
Supplement: Supplementary file 8 — Table S1. Main physicochemical characteristics of the three soils used in this study. [file MBT2-13-1648-s008.docx]

S1 Table. Main physicochemical characteristics of the three soils used in this study.

|  | Unit | Concentration ^*^ | | |
| --- | --- | --- | --- | --- |
|  |  | H | M | L |
| pH  Organic C  Total nitrogen (N)  C/N  Organic matter  CEC Metson  Calcium (Ca)  Magnesium (Mg)  Sodium (Na)  Potassium (K)  Iron (Fe)  Manganese (Mn)  Aluminium (Al)  Nitrate (NO_3_)  Ammonia nitrogen (NH_4_) | g.kg^-1^  g.kg^-1^  /  g.kg^-1^  cmol.kg^-1^  cmol.kg^-1^  cmol.kg^-1^  cmol.kg^-1^  cmol.kg^-1^  cmol.kg^-1^  cmol.kg^-1^  cmol.kg^-1^  mg.kg^-1^  mg.kg^-1^ | 6.21 ± 0.02  7.27 ± 0.16  0.77 ± 0.02  9.39 ± 0.03  12.60 ± 0.29  4.60 ± 0.11  4.19 ± 0.08  0.69 ± 0.01  0.0277 ± 0.0008  0.197 ± 0.004  0.0110 ± 0.0004  0.0180 ± 0.0003  0.073 ± 0.002  78.27 ± 0.64  3.26 ± 0.17 | 6.27 ± 0.01  7.05 ± 0.07  0.76 ± 0.01  9.31 ± 0.02  12.17 ± 0.11  4.48 ± 0.03  4.11 ± 0.02  0.67 ± 0.004  0.0248 ± 0.0004  0.199 ± 0.001  0.0099 ± 0.0001  0.0221 ± 0.0005  0.064 ± 0.002  69.37 ± 2.26  6.48 ± 1.19 | 6.87 ± 0.01  7.00 ± 0.09  0.74 ± 0.01  9.47 ± 0.02  12.13 ± 0.18  4.39 ± 0.13  3.94 ± 0.04  0.63 ± 0.01  0.0276 ± 0.0160  0.196 ± 0.002  0.0117 ± 0.0027  0.0167 ± 0.0007  0.069 ± 0.016  36.27 ± 1.46  25.97 ± 0.66 |

* Each value is the mean ± the standard error of three replicates

H, High diversity modality; M, Medium diversity modality; L, Low diversity modality.
